# Transcriptional signature of rapidly responding NK cells reveals S1P5 and CXCR4 as anti-tumor response disruptors

**DOI:** 10.1038/s41598-025-95211-7

**Published:** 2025-03-28

**Authors:** Marta Puig-Gámez, Martijn Van Attekum, Theodor Theis, Alec Dick, John E. Park

**Affiliations:** 1 Department of Cancer Immunology and Immune Modulation, Boehringer Ingelheim Pharma GmbH & Co. KG, 88387 Biberach an der Riss, Germany; 2 Department of Global Computational Biology and Digital Sciences, Boehringer Ingelheim Pharma GmbH & Co. KG, 88387 Biberach an der Riss, Germany; 3 Department of Medicinal Chemistry, Boehringer Ingelheim Pharma GmbH & Co. KG, 88387 Biberach an der Riss, Germany

**Keywords:** Immune evasion, Cancer immunotherapy, NK cells, Immunosuppression, Cancer immunotherapy, Cellular signalling networks

## Abstract

Natural killer (NK) cells are prototypic cytotoxic innate lymphocytes that can kill target cells, such as tumor cells, in the absence of antigen-restriction. Peripheral NK cells exhibit a high degree of heterogeneity. Here, we set out to broadly assess intrinsic modulators of NK cell degranulation in an unbiased manner. We stimulated human primary blood-borne NK cells pre-treated with different cytokine regimens with the HCT116 human colon cancer cell line and used detection of lysosome-associated membrane glycoprotein 1 (LAMP1) as an identifier of rapid NK cell degranulation. RNA sequencing of FACS-sorted LAMP1^hi^ NK cells showed *CXCR4* and *S1PR5* were top down-regulated genes. Using compounds that modulate activity of CXCR4 and S1P receptor family members S1P1 and S1P5, we confirmed they play an important immunosuppressive role in NK cell cytotoxicity. Mechanistically, engagement of CXCR4 and S1P1/5 receptors triggered phosphorylation of p42 and Ca^2+^ influx. CXCR4 activation promoted S1P5 upregulation and vice versa, and joint activation of both receptors amplified the defect NK cell degranulation. Intriguingly, in tumor samples the expression of both receptors and the synthesis of their ligands themselves appear to be coordinately regulated. Together, these data suggest that specifically and simultaneously targeting CXCR4 and S1P5 activity in the tumor microenvironment (TME) could be a beneficial strategy to unleash full cytotoxic potential of cytotoxic NK effector cells in the tumor.

**Supplementary Information:**

The online version contains supplementary material available at 10.1038/s41598-025-95211-7.

## Introduction

Natural Killer (NK) cells are cytotoxic lymphocytes specialized in the recognition and spontaneous killing of stressed cells^1–4^. While CD8 + T cell responses rely on MHC-I-mediated presentation of tumor-associated antigens, NK cells are triggered by the absence of MHC-I molecules in the plasma membrane^3,5^, a common occurrence in tumor cells^6^. Therefore, NK cell activity is critical for targeting tumor cells that escape killing by conventional CD8 + T cells. Indeed, infiltration of circulating NK cells in tumors is correlated with better prognosis in patients with a wide spectrum of solid tumors^7–10^. It has also been shown to protect patients against the development of tumor metastases^11^. However, there is still a significant gap in our understanding of what factors contribute to prime NK cells to detect and attack tumor cells. In fact, peripheral NK cells do not consistently infiltrate the tumor nor show the same ability to execute the cytotoxic program. Moreover, a fraction of NK cells lacks the receptors required to respond to stimulation with pro-inflammatory cytokines such as IL-2 or IL-15^12^. Similarly, although inhibition of NK cell activity with TGF-β will lead to a stark decrease in number of NK cells stimulated to degranulate by tumor cells^13^, a few outliers can remain sensitive to tumor cell challenge.

Stimulation of NK cells with tumor cells can lead to rapid degranulation and is an early indicator of cytotoxic effector cell activity. This process can be quantified by the detection of the lysosome-associated membrane glycoprotein 1 (LAMP1), also known as CD107a, in the plasma membrane. LAMP1 is otherwise normally only found in late endosomes and lysosomes^14 ^and in lytic granules in the case of cytotoxic effector cells^15^. Plasma membrane LAMP1 detection does not require fixation, takes place only upon stimulation and can be measured early on, making it a sensitive tool to identify recently activated NK cells. In this study, we set out to use LAMP1 detection as a tool to generate a transcriptional atlas of intrinsic modulators of circulating NK cell activation. We challenged human primary blood-borne NK cells treated with IL-2, TGF-β or a combination thereof, with the HCT-116 human colon cancer cell line. We used LAMP1 detection to distinguish NK cells poised to rapidly degranulate from non-responsive NK cells upon tumor cell challenge. We then performed bulk RNA-seq to identify the gene signatures underlying the differences between both populations. We hereby named the unique pattern of gene expression in LAMP1^hi^ NK cells the ‘Early Bird signature’. C-X-C Motif Chemokine Receptor 4 (*CXCR4*) and the Sphingosine-1-Phosphate Receptor 5 (*S1PR5*) stood out among genes that were most significantly repressed in degranulating cells. Interestingly, both CXCR4 and S1P5 have previously been shown to be involved in chemotaxis^16–18 ^and in maturation and egress of NK cells from the bone marrow^19,20^. To our knowledge, a function in NK cytotoxicity for either gene has not yet been reported.

Here, we identified a new role of S1P5, and more widely by the S1P receptor family, in the activation of NK cells. We also showed that CXCR4 binding by its cognate ligand CXCL12 leads to a defect in NK cell stimulation. Activation of S1P5 and CXCR4 triggered the same pathways downstream of the Gα_i_ protein and promoted signaling events akin to those triggered upon NK cell stimulation. Indeed, activation of S1P receptors with S1P agonist ceralifimod (ONO-4641) rendered NK cells less effective at subsequent tumor cell-dependent degranulation and killing, suggesting premature activation of NK cells through S1P receptors desensitizes cells to cytotoxic stimuli. Finally, we uncovered a positive correlation between CXCR4 and S1P5, wherein engagement of either receptor up-regulates the other, and their simultaneous activation additively promotes their immunosuppressive effect on NK cells.

## Results

**Fig. 1:**
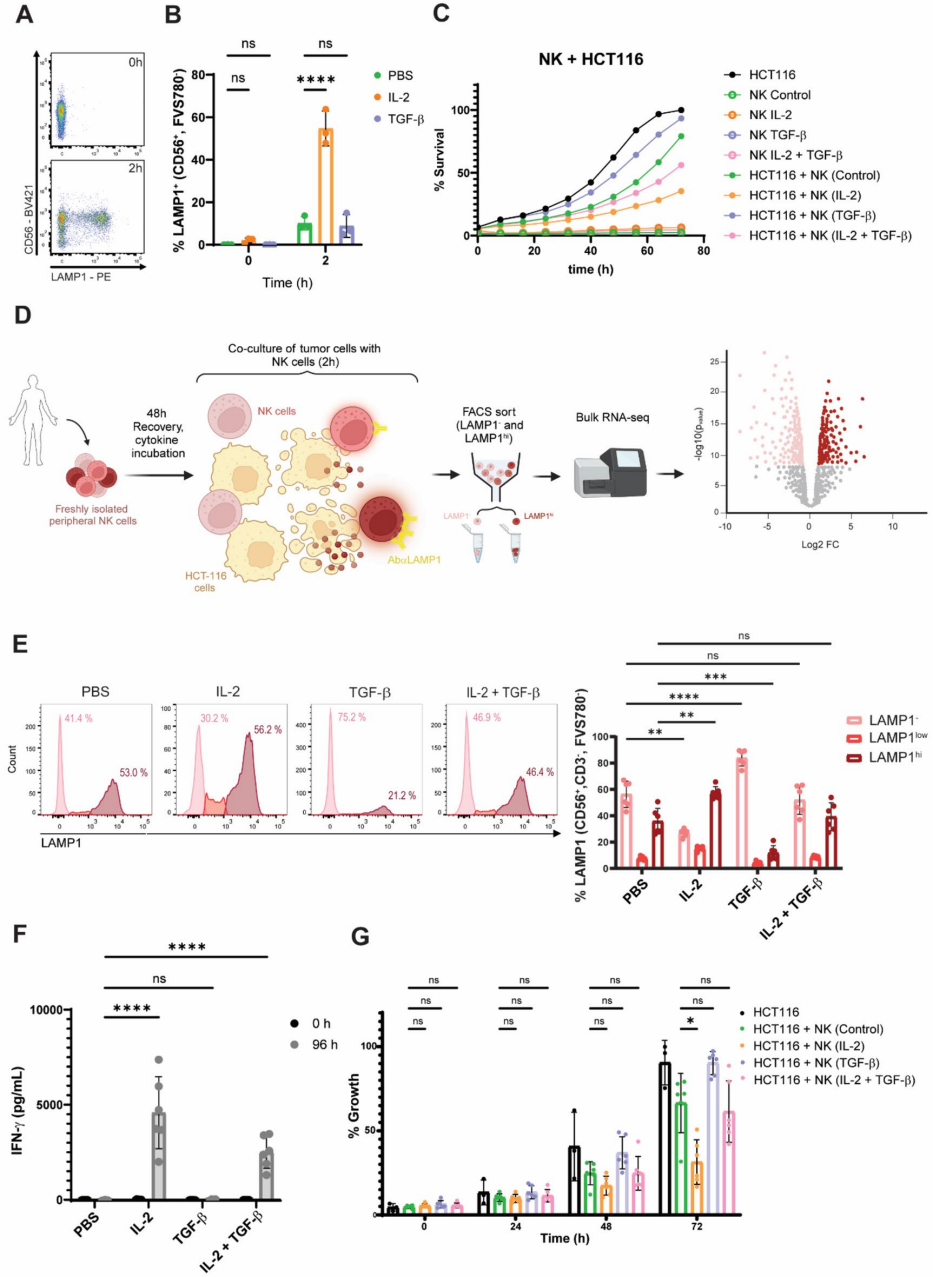
LAMP1 is detected at the cell surface of stimulated NK cells and can be modulated by IL-2 and TGF-β. In all instances, NK cells were treated for 48 h with the indicated cytokine regimens (IL-2 20 ng/mL, TGF-β 10 ng/mL or vehicle). For degranulation assays, pre-stimulated NK cells were co-cultured with HCT-116 at a 1:1 E: T ratio for 2 h. **A** Flow cytometry of LAMP1 levels in unstimulated (0 h) and HCT-116-challenged (2 h) NK cells. **B** Percentage of LAMP1^+^ cells in resting and HCT-116-challenged NK cells pre-treated with cytokine regimens. **C**) Survival of HCT-116 cells after 72 h co-culture with NK cells. **D** Graphic abstract of the strategy to identify new intrinsic modulators of NK cell activation created with BioRender.com. **E** Flow cytometry histograms of LAMP1 signal in cytokine-treated NK cells after stimulation with HCT-116 for 2 h (RNA-seq samples). **F** Secreted IFN-γ after 4 day co-culture of HCT-116 and NK cells. **G** Survival of HCT-116 cells after 96 h co-culture with NK cells. Signal was normalized to confluence in HCT-116 mono-culture wells. For all charts (**B**, **E–G**) every data point corresponds to the average value in each independent donor and bars the mean ± SD and Two-way ANOVA statistical tests were conducted. Dunnett’s test was applied for multiple comparisons except for the viability assessment (**G**), for which Tukey’s test was used. Symbols represent ns = not significant, *p-value < 0.05, **p-value < 0.01, ***p-value < 0.001, ****p-value < 0.0001.

### LAMP1 serves to distinguish dormant from responsive populations upon NK cell stimulation

To initiate NK cell anti-tumor cytotoxicity, the HCT-116 human epithelial cell line was used. HCT-116 cells express low levels of checkpoint inhibitor PD-L1 and MHC-I molecules which should trigger a strong NK cell anti-tumor response in vitro^21^. Indeed, while non-stimulated NK cells presented undetectable levels of LAMP1, stimulation of primary NK cells with HCT-116 in the presence of anti-human LAMP1 antibody led to the formation of a well-defined LAMP1^+^ population (Fig. 1A). This suggests degranulation only takes place upon stimulation of NK cells with tumor cells. Pre-incubation of NK cells with IL-2 and TGF-β resulted in a stark elevation and a slight reduction respectively in LAMP1 signal only when NK cells were stimulated with HCT-116 (Fig. 1B). In comparison to HCT-116, the SF-539 glioblastoma cell line expresses high levels of MHC-I molecules^21^. However, IL-2-treated NK cells still degranulated strongly once stimulated with SF-539 (Supplementary Fig. 1A). These results demonstrated the key role of IL-2 in NK cell activation. We then wanted to confirm that the changes in degranulation influenced NK cell cytotoxicity. NK cells pre-activated with IL-2 significantly reduced the extent of proliferation of HCT-116, as measured by cell confluence over time (Fig. 1C). Incubation with TGF-β had the opposite effect, and the combination resulted in intermediate values. All in all, LAMP1 was shown to be a marker present only upon stimulation and indicative of activated cytotoxic NK cells. In light of these data, we set out to use LAMP1 as a marker to identify rapidly responding NK cells to stimulation. We designed a workflow to assess differences between non-degranulating and degranulating cells’ gene expression patterns by bulk RNA-seq (Fig. 1D). Primary peripheral NK cells were obtained from the blood of 6 independent healthy donors and cultured for 48 h in media supplemented with PBS, IL-2, TGF-β or IL-2 and TGF-β co-treatment to minimize changes in gene expression in NK cells from in vivo to in vitro culture, whilst allowing cells to recover after selection. Cells were subsequently stimulated with HCT-116 for 2 h in the presence of LAMP1 antibody and sorted in two groups: non-responders (CD56^+^CD3^−^LAMP1^−^) or strongly degranulating cells (CD56^+^CD3^−^LAMP1^hi^) (Supplementary Fig. 1B). CD56^+^CD3^−^LAMP1^lo^ cells were left unsorted. 40,000 NK cells were analyzed per condition and LAMP1 score, unless otherwise indicated (Supplementary Fig. 1C). NK cell viability was not compromised in the absence of these cytokines (Supplementary Fig. 1D). As expected, LAMP1 levels (Fig. 1E, Supplementary Fig. 1E), interference with IFN-γ secretion (Fig. 1F) and blockade of HCT-116 proliferation in co-cultures (Fig. 1G) were higher in samples from NK-cells treated with IL-2 and significantly reduced in the TGF-β-treated samples. Altogether, our data suggest that degranulation, as measured by LAMP1 detection, can be used as a marker of early stimulation and is subject to the activation status of NK cells.

**Fig. 2:**
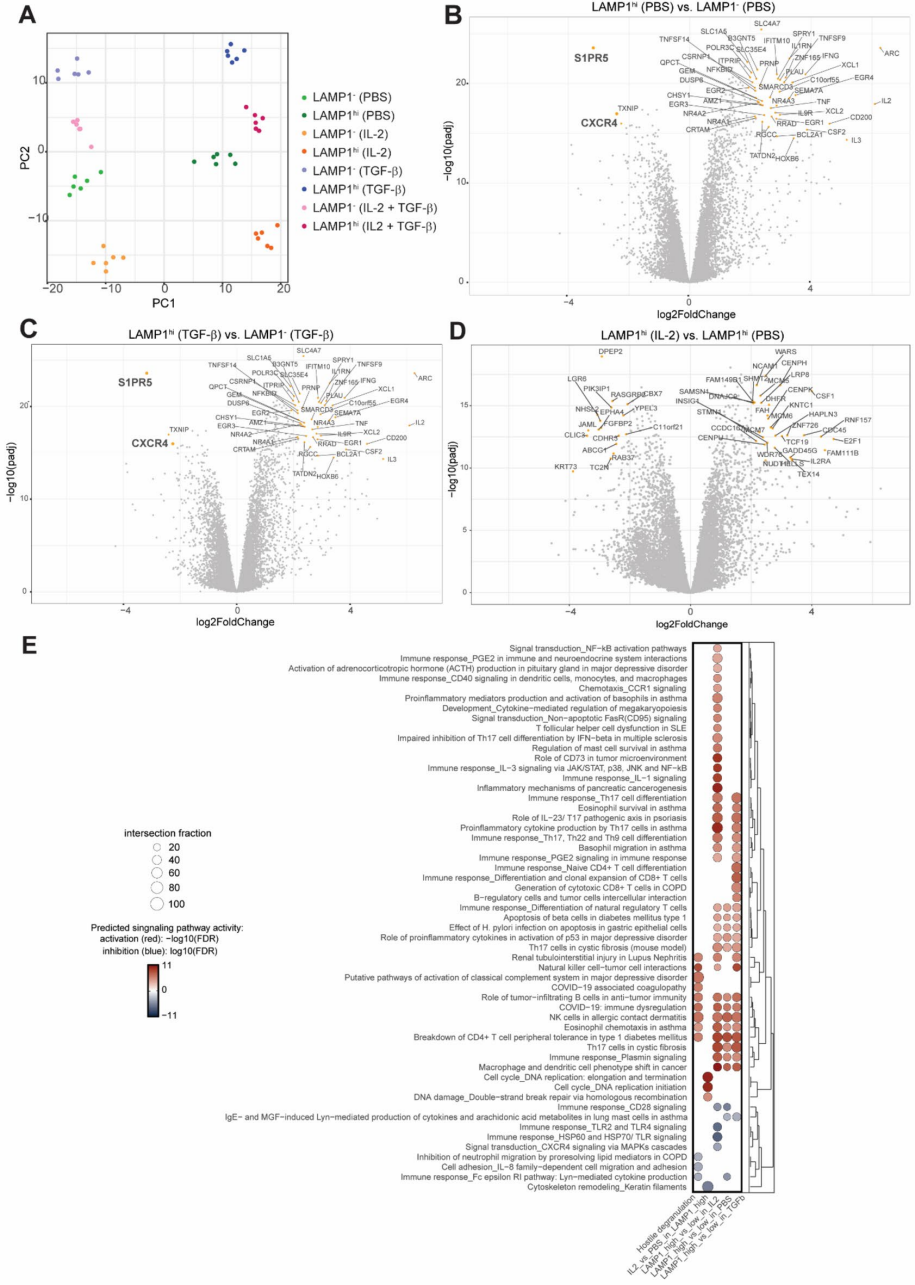
Bulk RNA-seq produced a comprehensive list of differentially regulated genes in LAMP1^**−**^ and LAMP1^hi^ NK cells. **A** Principal Component (PC) analysis plot based on gene expression values. Every point represents a sample belonging to an independent donor. Volcano plots of genes differentially expressed in LAMP1^hi^ against LAMP1^−^ cells in untreated NK cells (PBS) (**B**), LAMP1^hi^ versus LAMP1^−^ cells in cells treated with TGF-β (**C**) and LAMP1^hi^ cells treated with IL-2 versus vehicle (**D**). Genes highlighted in orange pertain to the top 50 differentially regulated genes according to log2Fold Change and -log10 adjusted p-value (-log10(padj)) in each comparison. **E** Dot plot of significantly differentially regulated genes in each comparison. Top 100 pathways across all comparisons were selected based on perturbation (effect size) and adjusted p-value. Degree of activation of each pathway is indicated by color of the dots and their size illustrates the fraction of intersection with genes included in each pathway.

### LAMP1^hi^ NK cells exhibit a clear transcriptional signature characterized by genes involved in inflammation and cytotoxicity

Principal Component Analysis (PCA) of expression data of RNA-seq samples showed samples tended to cluster by treatment and LAMP1 score (Fig. 2A), demonstrating the robustness of our assay. Three main comparisons of samples were further analyzed to gain a deeper understanding into factors that modulate NK cell activity, for which volcano plots were generated. Only the top 50 differentially regulated genes were annotated in the volcano plots to allow clear visualization. First, LAMP1^hi^/LAMP1^−^ cells were compared in the vehicle condition to gain a general understanding of which factors modulate NK cell stimulation and degranulation (Fig. 2B). The comparison of TGF-β-LAMP1^hi^/TGF-β-LAMP1^−^ was used to identify those genes expressed in cells which successfully execute degranulation, even in hostile conditions, (Fig. 2C). Finally, the IL-2-LAMP1^hi^/PBS-LAMP1^hi^ comparison identifies genes that are upregulated by IL-2 treatment that support degranulation of otherwise inactive cells (Fig. 2D). To gain a deeper understanding of which biological processes are enriched in these comparisons, we additionally performed a gene enrichment pathway analysis (Fig. 2E). A great number of pathways were enriched in all comparisons of LAMP1^hi^/LAMP1^−^ samples regardless of treatment. This group included several pathways involved in NK cell function and general immune cell signaling in response to disease. Interestingly, the top enriched pathways in the IL-2 vs. PBS LAMP1^hi^ column were unique to this comparison, and were all related to DNA replication and repair, consistent with reports that resting T cells are hypersensitive to DNA damage^22^. Finally, the TGF-β vs. PBS LAMP1^hi^ comparison showed an overlap in activated pathways with LAMP1^hi^/LAMP1^−^comparisons, but also inactivation of select pathways important for cell adhesion and migration, in line with other studies exploring the role TGF-β on NK cell function^23^.

To interrogate the role of individual genes in NK cell function, we turned our attention to differentially regulated genes in the LAMP1^hi^/LAMP1^−^ PBS (control) comparison. Only 3 out of the top 50 differentially regulated genes were downregulated in LAMP1^hi^ cells: *S1PR5*, *TXNIP* and *CXCR4*. Interestingly, they stood out both in the control comparison but also as genes highly expressed in non-degranulating cells in the TGF-β condition. *TXNIP *encodes the thioredoxin-interacting protein, which interacts with and blocks thioredoxin antioxidant function, thus playing a key role in cellular redox homeostasis. It has also been reported to be important for NK cell development and to inhibit IFN-γ production of NK exposed to bacterial infection^24,25^. CXCR4 signaling via MAPKs cascades was one of the few pathways inhibited in the LAMP1^hi^/LAMP1^−^ comparisons in the gene enrichment pathway analysis, further supporting a role of CXCR4 and its signaling cascade in degranulation. Intriguingly, both *CXCR4* and *S1PR5 *are intimately related as key components of the maturation and egress of NK cells from lymphoid organs^19^. However, expression of these proteins is carried on in peripheral NK cells, suggesting these proteins may play other functions once NK cells are released into the bloodstream^26^. We thus aimed to explore the putative function of *CXCR4* and *S1PR5* in NK cell cytotoxicity.

**Fig. 3:**
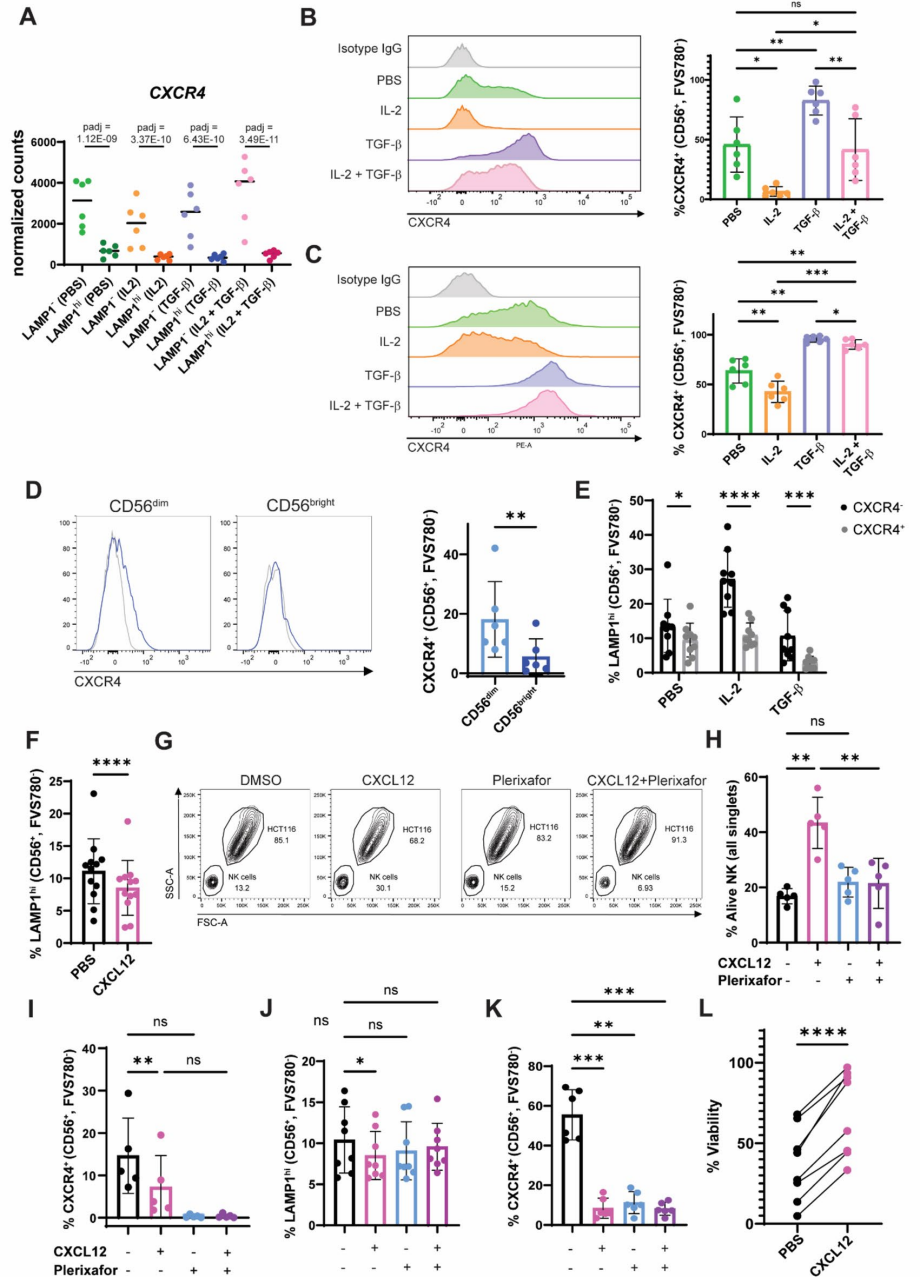
Activation of CXCR4 hinders degranulation in stimulated NK cells. **A** *CXCR4* expression quantified as normalized counts in the conditions used for RNA-seq. Flow cytometry quantification of CXCR4 signal in NK cells treated with IL-2, TGF-β or combination cytokine regimens at high (10 ng/mL) (**B**) or low concentration (0.5 ng/mL) (**C**) after 48 h of treatment. **D** Flow cytometry histograms indicating levels of CXCR4 at the cell surface of CD56^dim^ and CD56^bright^ freshly isolated human peripheral NK cells. **E** Percentage of LAMP1^hi^ cells in CXCR4^−^ and CXCR4^+^ NK populations after co-culture with HCT-116 for 2 h following 48 h of cytokine regimens. **F** Degranulation triggered by 2 h HCT-116 stimulation of NK cells after overnight culture with 100 ng/mL CXCL12 or vehicle. **G**) Density plots of cell types present in the lower chamber of a transwell plate in a migration assay. NK cells were placed in the upper chamber and left to migrate for 2 h towards HCT-116 cells in the lower chamber in the presence or absence of a 100 ng/mL CXCL12 gradient. NK cells were pre-treated with 10 µM plerixafor or vehicle for 2 h prior to transfer to transwell plate. **H** Percentage of viable NK cells out of total single cells in the lower chamber. **I** Percentage of CXCR4^+^ NK cells in the lower chamber of the transwell plate at the end of the experiment. Degranulation of NK cells (**J**) and CXCR4 levels (**K**) after 2 h co-culture with HCT-116 following overnight NK cell treatment with CXCL12 and plerixafor. **L** Cell survival of HCT-116 cells after 24 h of co-culturing with NK cells previously treated with indicated regimens. For all charts, every data point corresponds to the average value in each independent donor and bars the mean ± SD. Adjusted p-values (padj) were calculated using DESeq for RNA-seq gene expression analysis (**A**). T student tests were used for comparisons between two conditions (**D**, **F**,** L**). One-way ANOVA statistical tests were used in systems with several conditions for a single variable (**B**, **C**, **H**, **I**, **J**, **K**). For these, Dunnett’s test (**B**, **C**, **H**, **I**) or Tukey’s test (**I**, **J**) were used to perform multiple comparisons. The Two-way ANOVA statistical test was performed for analysis containing two variables (**E**), for which Šídák’s test for multiple comparisons was used. Symbols represent ns = not significant, *p-value < 0.05, **p-value < 0.01, ***p-value < 0.001.

### The CXCR4-CXCL12 chemokine signaling axis hampers NK cell stimulation by tumor cells

We first sought to characterize cell surface CXCR4 in NK cells. *CXCR4* was highly expressed in dormant NK cells, but markedly downregulated in degranulating cells (Fig. 3A). Additionally, IL-2 treatment reduced the level of *CXCR4* expression, which translated into a lower CXCR4 availability at the plasma membrane and might thus sensitize the cells to degranulate (Fig. 3B**)**. Conversely, incubation with TGF-β rendered most NK cells CXCR4^+^, suggesting a link to an NK cell immunosuppressive phenotype. At low concentrations, the effect by TGF-β was comparatively stronger than IL-2, as it could rescue CXCR4 expression (Fig. 3C). In comparison to NK cells, the other main cytotoxic effector cell lineage, CD8^+^ T cells, remained mostly CXCR4^+^ in all conditions tested (Supplementary Fig. 2A). Levels of CD56 in the cell surface have been shown to be indicative of the maturity state of human NK cells; CD56^bright^ are considered to be a less mature, immunoregulatory subset while CD56^dim^represent a more terminally differentiated cytotoxic NK cell compartment^27^. Most CXCR4^+^ NK cells were CD56^dim^ (Fig. 3D), suggesting cell immaturity does not underly the reduced degranulation in CXCR4 expressing cells. In vitro culture of NK cells with IL-2 and subsequent stimulation with HCT-116 lead to a stark increase in degranulation in CXCR4^−^ but not CXCR4^+^ subsets (Fig. 3E), an effect also observed in CD8^+^ T cells (Supplementary Fig. 2B). Interestingly, in the TGF-β treatment, the few remaining degranulating cells were strictly CXCR4^−^. Collectively, these data suggest that CXCR4 expression correlates to lower responsiveness to stimulation by tumor cells.

CXCR4 is a chemokine receptor that selectively binds the stromal cell-derived factor (SDF-1), also known as CXCL12. This receptor is best known for its role in homing NK and CD8^+^T cells in the bone marrow and orchestrating migration towards CXCL12-rich environments^28^. We next sought to investigate if CXCL12 could additionally play a role in NK cell activation. To do this, we measured degranulation in CXCL12-treated or untreated NK cells stimulated with HCT-116. Pre-treatment with CXCL12 resulted in a significant reduction of the LAMP1^hi^ population (Fig. 3F), suggesting an active role by CXCR4 in suppressing NK cell activity. We ruled out the possibility of this effect being mediated by the other binding partner of CXCL12, as CXCR7-encoding *ACKR3 *is barely expressed in peripheral NK cells (Supplementary Fig. 2C). Plerixafor (AMD3100) is a CXCR4 inhibitor that induces receptor internalization through β-arrestin recruitment while fully antagonizing CXCR4^29^. As expected, pre-incubation of NK cells with plerixafor blocked CXCL12-dependent chemotactic migration of NK cells towards HCT-116 in a transwell assay (Fig. 3G-H) without affecting NK cell viability (Supplementary Fig. 2D). Plerixafor did not exert an effect in the absence of CXCL12. This could be explained by the lack of detectable CXLC12 in supernatants from HCT-116, NK cells, CD8^+^ T cells or combined cultures of both cell types in vitro (Supplementary Fig. 2E-2 F). Interestingly, exposure for two hours to CXCL12 was sufficient to observe a reduction in CXCR4 levels in migrated NK cells, suggesting rapid internalization of the receptor upon engagement (Fig. 3I). We then examined whether plerixafor could similarly block the effect of CXCL12 in degranulation. Indeed, co-treatment of NK cells with CXCL12 and plerixafor rescued the degranulation defect observed by incubation with CXCL12 alone (Fig. 3J), while inducing CXCR4 internalization to a similar extent as the ligand (Fig. 3K). In contrast, although CXCL12 promoted internalization of CXCR4 in both CD8^+^ T cells (Supplementary Fig. 2G), it had the opposite effect to NK cells in CD8^+^ T cell degranulation (Supplementary Fig. 2H). However, in T cells, no significant differences were observed in killing assays when CD8 + T were stimulated in the presence of HCT-116 with a bispecific EpCam T cell engager (Supplementary Fig. 2I). The NK cell killing assay was performed in NK cells pre-activated with IL-2 in order to induce sufficient measurable killing. Although IL-2 reduces CXCR4 levels in the membrane (Supplementary Fig. 2J), a moderate reduction in killing of HCT-116 concomitant to the defect observed in degranulation was detected by NK cells pre-treated with CXCL12 in comparison to the control (Fig. 3L).

Taken together, these findings suggest that the CXCL12/CXCR4 axis has immune suppressive activity in NK cells, which may play a complementary role to its function in migration.

**Fig. 4:**
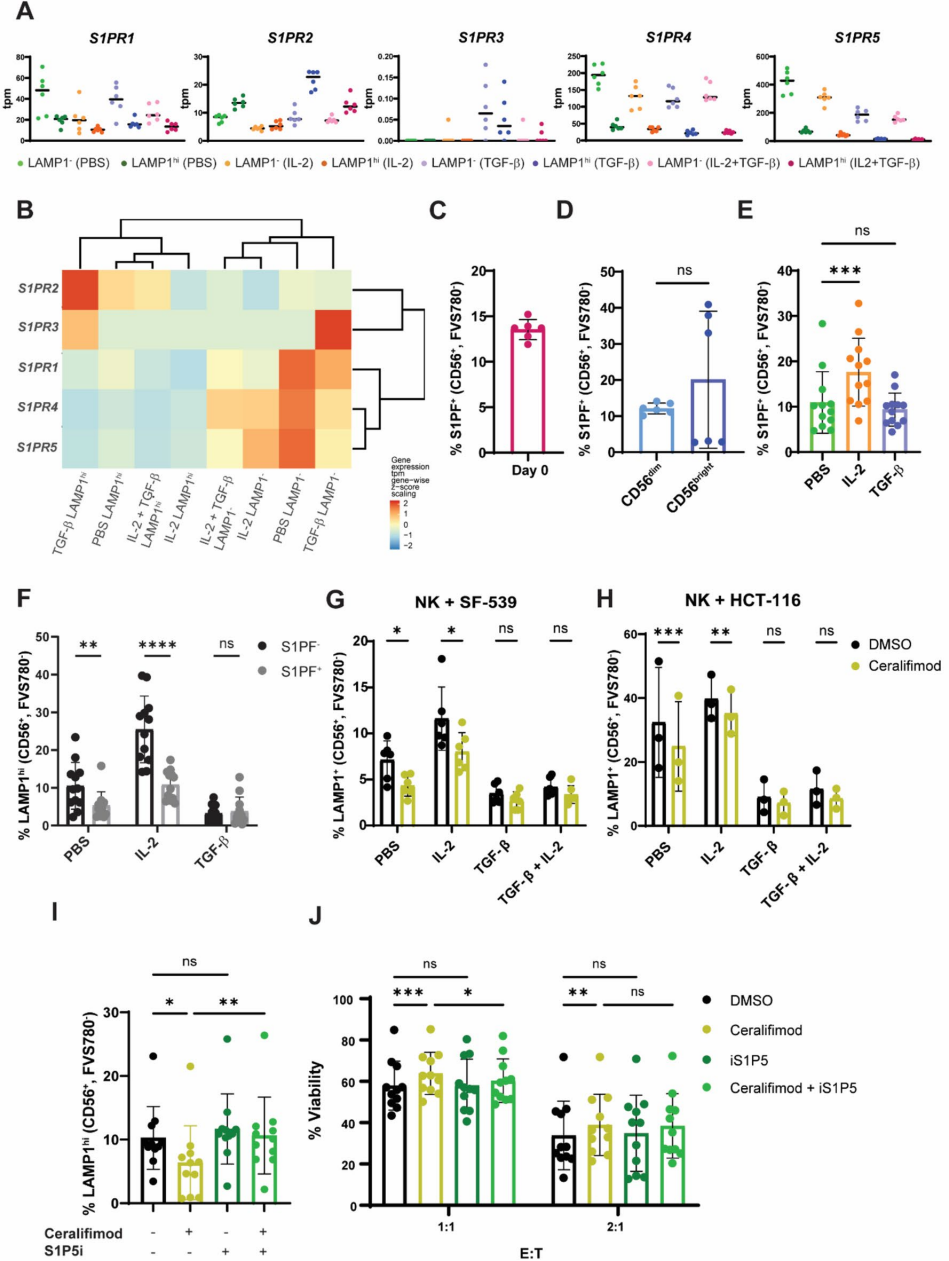
NK cells pre-treated with S1P1/5 agonist ceralifimod are less reactive to stimulation by tumor cells. **A** S1P receptor family gene expression quantified in transcripts per million (tpm) in the RNA-seq. **B** Heat map showing scaled expression of S1P receptor genes in each treatment and LAMP1 classification of NK cells. **C** Percentage of freshly isolated NK cells binding to S1PF. **D** Flow cytometry indicating levels of S1P fluorescein binding to CD56^dim^ and CD56^bright^ freshly isolated human peripheral NK cells. **E** Quantification of S1PF binding to NK cells treated with cytokine regimens (10 ng/mL IL-2, 10 ng/mL TGF-β or vehicle) 2 days after treatment. **F** Percentage of LAMP1^hi^ cells in S1PF^−^ and S1PF^+^ NK populations after co-culture with HCT-116 for 2 h following 48 h of cytokine treatment. Percentage of LAMP1^+^ NK cells upon 48 h treatment with IL-2 in combination with 1 µM ceralifimod or DMSO and stimulation with SF-539 (**G**) or HCT-116 (**H**) cells for 2 h. **I** Percentage of LAMP1^+^ NK cells after co-culture with HCT-116 for 2 h following 48 h of cytokine, 1 µM ceralifimod and 1 µM inhibitor regimens. **J** Survival of HCT-116 cells after 24 h of co-culturing with NK cells previously treated with indicated regimens at different E: T ratios. E:T stands for Effector:Tumor cell. For all charts, every data point corresponds to the average value in each independent donor and bars the mean ± SD. T student tests were used for comparisons between two conditions (**D**). One-way ANOVA statistical tests were used in systems with several conditions for a single variable (**E**, **I**). Dunnett’s test was used to perform multiple comparisons. The Two-way ANOVA statistical test was performed for analysis containing two variables (**F**, **G**, **H**, **J**), for which Šídák’s test (**F**, **G**, **H**) or Dunnett’s test (**J**) for multiple comparisons were used. Symbols represent ns = not significant, *p-value < 0.05, **p-value < 0.01, ***p-value < 0.001, ****p-value < 0.0001.

### Activation of S1P1/5 with Ceralifimod dampens NK cell cytotoxic response

Next, we investigated the function of *S1PR5* in NK cell cytotoxicity. *S1PR5 *encodes S1P5, the fifth member of the sphingosine-1-phosphate (S1P) receptor family. S1P receptors are G protein-coupled receptors (GPCRs) that trigger G protein signal transduction upon binding to the bioactive lipid S1P. Expression of these receptors is rather ubiquitous, with specific patterns depending on the cell type^30^. Peripheral NK cells expressed mainly *S1PR4* and *S1PR5*, followed by modest expression of *S1PR1* and low *S1PR2* (Fig. 4A). Interestingly, *S1PR1*, *S1PR4* and *S1PR5* were all downregulated in LAMP1^hi^ cells in comparison to non-responders. Indeed, *S1PR1*, *S1PR4* and *S1PR5* expression clustered together in all the conditions included in the RNA-seq, suggesting a positive correlation in the expression of these receptors, in contrast to *S1PR2* and *S1PR3* (Fig. 4B). Of note, although *S1PR5* scored the highest, *S1PR4 *was among the top 30 genes downregulated in rapidly responding NK cells in our RNA-seq data (Supplementary Data Table 1). Considering the higher levels of expression and the functional redundancy of S1P1, S1P4 and S1P5 signaling pathways^30^, we used fluorescein-conjugated S1P (S1PF) to examine the presence of all S1P receptors in the cell surface. S1PF binding recapitulated S1P, as shown by loss of S1PF detection in cells previously blocked with unconjugated lipid (Supplementary Fig. 3A). Surprisingly, the frequency of S1PF^+^ cells was only 12–15% in peripheral NK cells (Fig. 4C). S1P binding is known to cause S1P receptor internalization and subsequent recycling to the cell surface^31,32^. Blood is the richest body tissue in S1P levels (100–400 nM)^33^. Therefore, blood-borne S1P could be responsible for the low yield of S1PF-binding peripheral NK cells, and S1P in fetal calf serum used to supplement NK cell medium (Supplementary Fig. 3B) presumably affects receptor dynamics. There was no direct correlation between CD56 levels and S1PF binding to the cells (Fig. 4D), suggesting S1P receptor expression does not rely on maturity of NK cells. Interestingly, no S1PF binding was detected in peripheral CD8^+^ T cells (Supplementary Fig. 3C). This was in line with *S1PR5 *gene expression data extracted from transcriptomic studies in immune cells^34^ (Supplementary Fig. 3D). The S1PF^+^ NK cell subset expanded in response to IL-2 (Fig. 4E), together with S1P1^+^ (Supplementary Fig. 3E), but S1PF^+^CD8^+^ T cells remained undetectable (Supplementary Fig. 3F). These results suggest that S1P-mediated signaling is not a relevant mechanism for circulating CD8^+^ T cells. We then examined degranulation in the S1PF subsets. As observed during examination of the CXCR4 receptor, S1PF^−^ cells degranulated significantly better than S1PF^+^, supporting the presence of S1P receptors as a sign of NK cell dormancy (Fig. 4F).

Next, we asked if S1P receptor activation directly impacts NK cell activity. We used ceralifimod, a second-generation S1P receptor agonist selective for S1P1 and S1P5^35^. Pre-treatment with ceralifimod significantly reduced the extent of degranulation in NK cells challenged with SF-539 (Fig. 4G) and HCT-116 cells (Fig. 4H), while S1PF binding and S1P1 levels remained largely unaffected (Supplementary Fig. 3G-3 H). As expected, ceralifimod did not impact CD8^+^T cell degranulation (Supplementary Fig. 3I). The defect in degranulation by NK cells could be blocked by co-treating the cells with a previously described highly specific S1P5 inhibitor (S1P5i)^36^ (Fig. 4I), which restored the numbers of the LAMP1^hi^ population at baseline. Neither S1P5i nor ceralifimod had any effect on NK cell viability (Supplementary Fig. 3J). To further decipher the effect of ceralifimod in NK cells, we challenged NK cells with SF-539 and HCT-116 cells and monitored tumor cell viability after 24 h. Consistent with our observations in degranulation, SF-539 cells were highly resistant to NK cell killing and did not succumb to NK cell attack in any condition (Supplementary Fig. 3K). On the contrary, HCT-116 cells elicited a strong NK cell response, which was significantly dampened in NK cells pre-treated with ceralifimod (Fig. 4J). Surprisingly, S1P5i only partially restored NK cell killing when ceralifimod and ceralifimod + S1P5i conditions were compared. This suggests S1P1 is partly responsible for the effect of ceralifimod in the inhibition of NK cell activity. Together, these results point at an immunosuppressive role of S1P1 and S1P5 in anti-tumor NK cell activity.

**Fig. 5:**
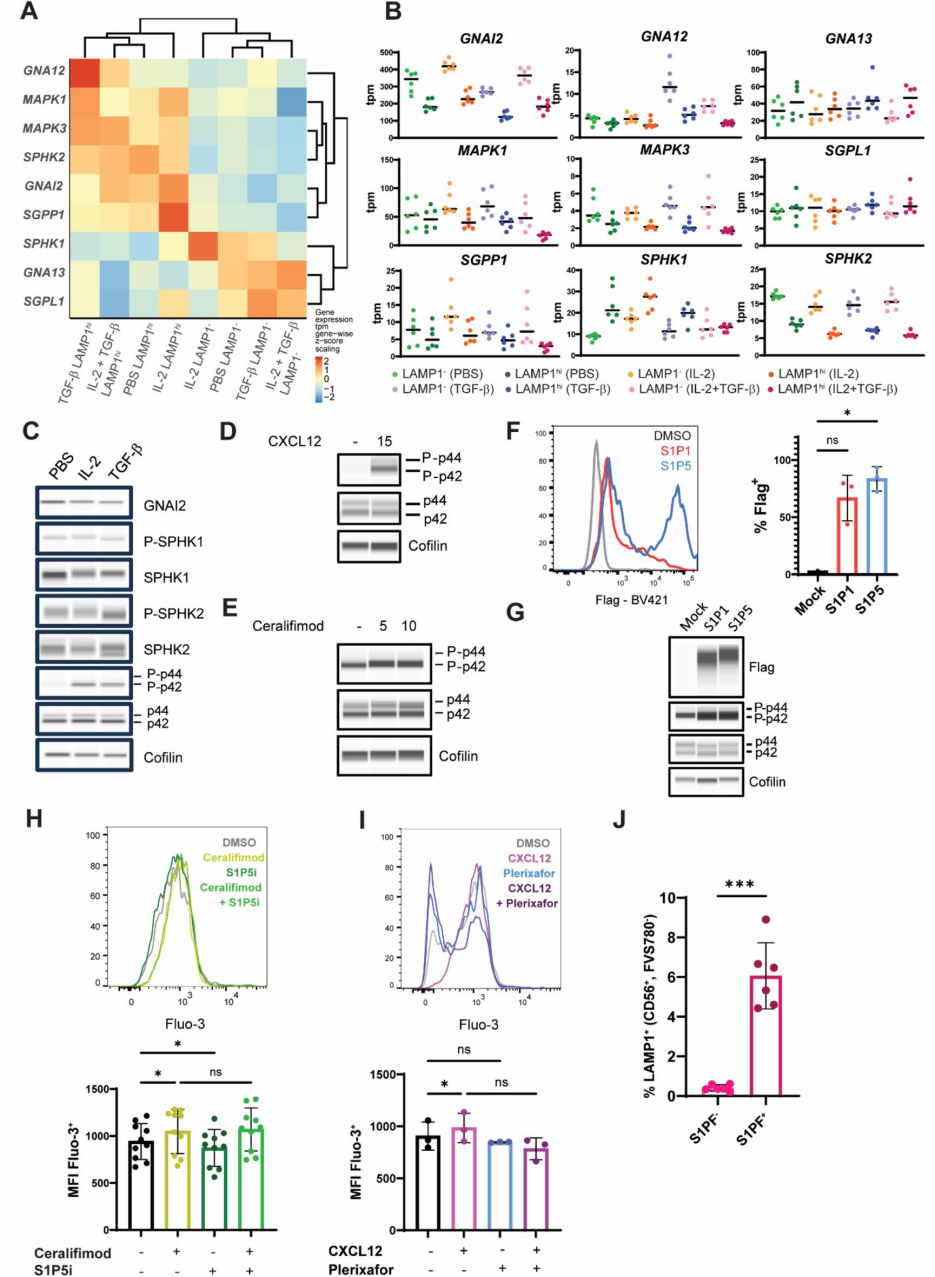
CXCL12 and ceralifimod signal through Gα_**i**_. **A** Heat map showing scaled expression of proteins in the S1P receptor signaling cascade. **B** Gene expression of selected genes involved in S1P signaling quantified in transcripts per million (tpm). **C**) Immunoblot of selected proteins in the S1P receptor signaling pathway for NK cells treated with vehicle, IL-2 and TGF-β for 48 h. Phosphorylation of p42 and p44 proteins upon stimulation of NK cells with 100 ng/mL CXCL12 for 15 min (**D**) or 1 µM ceralifimod for 5 and 10 min (**E**). **F** Representative histogram and quantification of Flag-tag signal in HCT-116 transfected with Flag-S1P1 and Flag-S1P5 encoding plasmids. **G** Protein detection of p42 phosphorylation in HCT-116 cells that overexpress Flag-S1P1/5 proteins. Flow cytometry and quantification of NK cells treated overnight with 1 µM ceralifimod and 1 µM S1P5i (**H**) or 100 ng/mL CXCL12 and 10 µM plerixafor (**I**) regimens. (**J**) Detection by flow cytometry of LAMP1^+^ cells within the S1PF^−^ and S1PF^+^ resting NK cell subsets. T-student test was performed to assess statistical significance. Immunoblots show data for a representative from a total of three. For immunoblots, images were cropped to show the approximate expected molecular size of the protein. For all charts, every data point corresponds to the average value in each independent donor and bars the mean ± SD. One-way ANOVA statistical tests were used in systems with several conditions for a single variable (**F**, **H**, **I**). For these, Dunnett’s test was used to perform multiple comparisons. Symbols represent ns = not significant, *p-value < 0.05, ***p-value < 0.001.

### Gα_i_ signaling triggered by CXCR4 and S1P1/5 promotes pathways involved in NK cell activation

To further understand the mechanisms underlying the inhibitory effect of ceralifimod, we extended the characterization of the NK cells used for RNA-seq. We explored the expression proteins involved in the metabolism of S1P, including sphingosine kinase 1 (*SPHK1*) and 2 (*SPHK2*), S1P phosphatase (*SGPP1*) and S1P lyase (*SGPL1*), which irreversibly degrades S1P (Fig. 5A). No changes in *SGPL1* were detected across conditions. However, while *SPHK2* and *SGPP1* were downregulated in LAMP1^−^ samples, *SPHK1 *followed the opposite pattern. It has been shown by others that SPHK1 and SPHK2 proteins have opposing functions^37^. SPHK1 is present in the cytosol and docks to the inner leaflet of the plasma membrane when activated. The S1P thus produced can either directly execute functions in the cytosol or be transported through specialized channels and signal in an autocrine “inside-out” fashion via S1P receptors. Conversely, SPHK2 localization is restricted to the nucleus, and its function antagonizes that of SPHK1, favoring a more pro-apoptotic phenotype in comparison to the protective effect exerted by SPHK1 against stress-induced cytotoxicity^37,38^. We additionally investigated expression of the three G proteins S1P4 and S1P5 can interact with, Gα_i_ (*GNAI2*), Gα_12_ (*GNA12*) and Gα_13_ (*GNA13*). *GNAI2* and *GNA12* negatively correlated to LAMP1 signal in a similar way to *S1PR1*, *S1PR4* and *S1PR5*. However, *GNAI2* was upregulated several fold in comparison to *GNA12* and *GNA13*, and showed the clearest differences between LAMP1^−^ and LAMP1^hi^ conditions (Fig. 5B). Protein and phosphorylation levels of all kinases above could be detected by immunoblot, indicating the observations at the level of mRNA also translated into protein (Fig. 5C). These data, together with the correlation observed between *GNAI2* and *MAPK1* and *MAPK3* expression (Fig. 5A), indicate that the S1P signaling pathway is more generally implicated in NK cell activity, and suggest that peripheral NK cells may primarily signal through Gα_i_ upon S1P receptor engagement.

Interestingly, CXCR4 has itself been reported to signal through Gα_i_^39^. We thus set to examine whether CXCL12 and ceralifimod may trigger the same signaling pathway. Indeed, short-term exposure of NK cells to CXCL12 and ceralifimod led to an increase in phosphorylation levels of p-42 (Fig. 5D, E). In comparison to CXCL12, ceralifimod only triggered a small increase in p42 phosphorylation, likely due to the smaller subset of S1PF^+^ NK cells in contrast to CXCR4^+^. To confirm that S1P receptors can potently induce p42 phosphorylation, we transfected HCT-116 cells with plasmids encoding Flag-tagged S1P1 and S1P5 (Fig. 5F). Media used for HCT-116 culture contains serum and thus, S1P ligand, making expression of S1P receptors sufficient to trigger a strong phosphorylation of p42 (Fig. 5G). Together, these data confirmed that p42 is activated downstream of CXCR4 and S1P1/5 binding to their cognate ligands. Another downstream event of Gα_i_ activation is the rise in cytosolic Ca^2+^. Indeed, an increase in intracellular Ca^2+^ was observed upon treatment of NK cells with ceralifimod (Fig. 5H) and CXCL12 (Fig. 5I), which was blocked when used in combination with S1P5i and plerixafor respectively. Interestingly, S1P5i appeared to reduce to an extent cytosolic Ca^2+^ on its own, likely by blocking signaling of natural S1P present in the culture media. Overall, these findings indicate that CXCR4 and S1P1/5 promote Gα_i_ signaling. Interestingly, both p42 phosphorylation and Ca^2+^are important biological processes required for NK cell cytotoxicity. p42 (also known as ERK2) is involved in the successful polarization of lytic granules^40^, while Ca^2+^influx is critical for vesicle release during degranulation^41^. We speculated that sub-chronic pre-exposure of NK cells to triggers of Gα_i_ signaling can result in NK cell desensitization to stimulation induced by tumor cells. To explore this hypothesis, we investigated the small percentage of LAMP1^+^ NK cells occurring in the absence of stimulation with tumor cells. In this context, all LAMP1^+^ cells bound S1PF (Fig. 5J). This could be explained by the interaction of S1P receptors with media-derived S1P initiating a low-level activation in NK cells, which in few instances results in successful degranulation. Collectively, our data points at CXCR4 and S1P1/5 as initiators of low-grade stimulation that partially desensitizes NK cells to productive stimulation by tumor cells.

**Fig. 6:**
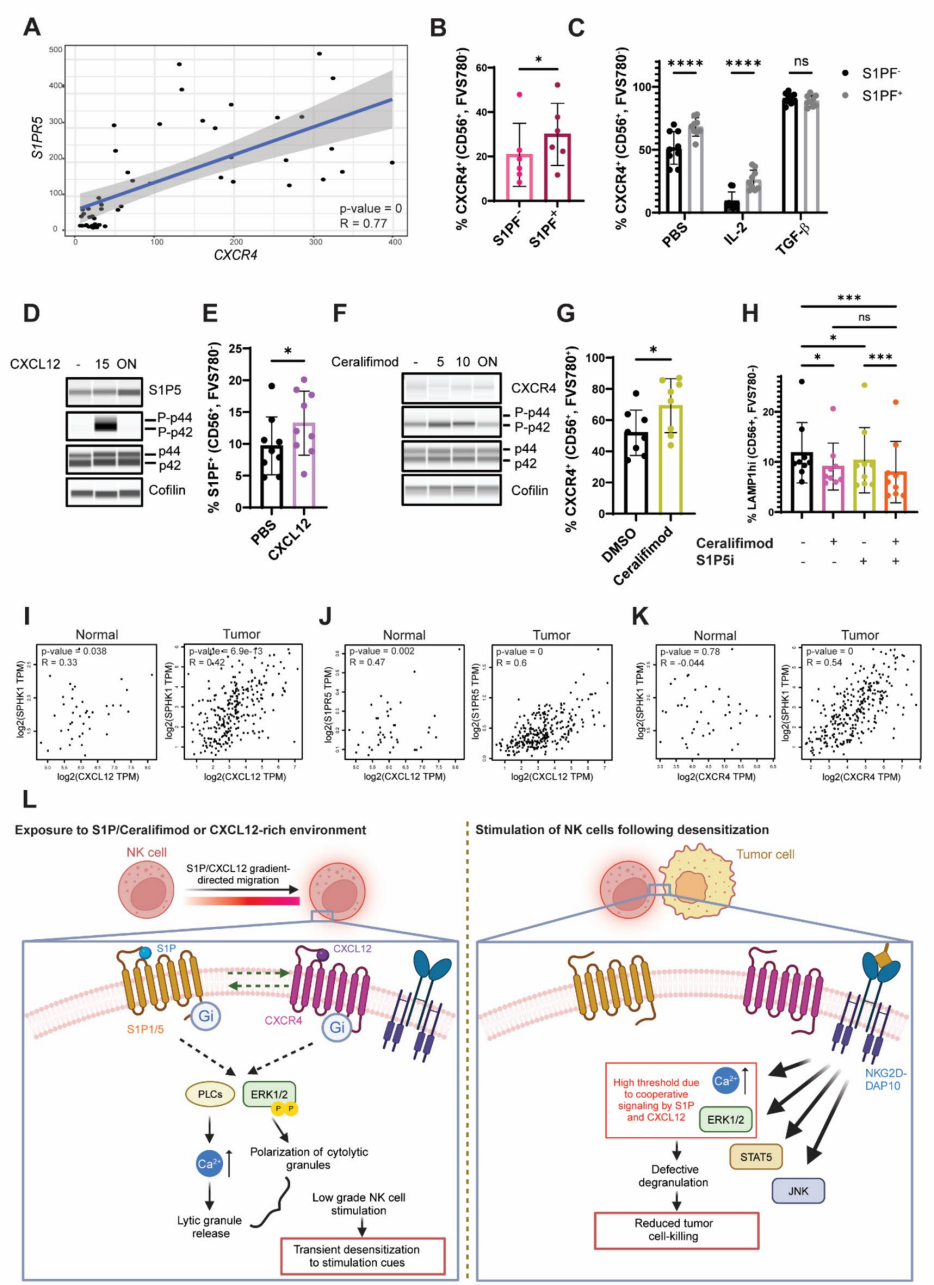
Activation of CXCR4 and S1P1/5 drive upregulation of one another and maximize defects in NK cell degranulation. **A** Correlation *CXCR4* and *S1PR5* expression in NK cells used for RNA-seq. Percentage of CXCR4^+^ cells in S1PF^−^ and S1PF^+^ NK cell subsets at the day of isolation (**B**) or after 48 h treatment with IL-2, TGF-β or vehicle (**C**). **D** Western blot of NK cells subjected to 100 ng/mL CXCL12 for 15 min or overnight culture. **E** Detection of S1PF binding in rested and CXCL12-treated NK cells. **F** Immunoblot of NK cells treated with 10 ng/mL IL-2 for 48 h different periods of 1 µM ceralifimod exposure. **G** Detection of CXCR4 in rested and ceralifimod-treated NK cells. **H** NK cell degranulation measured by LAMP1^hi^ detection after treatments with CXCL12, ceralifimod or ceralifimod + CXCL12 followed by stimulation with HCT-116 for 2 h. Correlation between gene expression of the pairs: *CXCL12* + *SPHK1* (**I**), *CXCL12* + *S1PR5* (**J**) and *CXCR4* + *SPHK1* (**K**) in normal tissue and tumor biopsies from patients suffering from colon adenocarcinoma. Values were obtained from The Cancer Genome Atlas (TCGA) and plotted using GEPIA 2^42^. Linear correlation was measured using the Pearson coefficient. **L** Schematic model showing the mechanism of CXCR4 and S1P1/5 desensitization of NK cells (created with BioRender.com). Immunoblots show data for a representative donor from a total of three. For immunoblots, images were cropped to cover the expected molecular size of the protein. For all charts, every data point corresponds to the average value in each independent donor and bars the mean ± SD. T student tests were used for comparisons between two conditions (**C**, **E**, **G**). One-way ANOVA statistical test was used in systems with several conditions for a single variable (**H**) and Two-way ANOVA was used when two variables were assessed (**C**). For both, Šídák’s test was used to examine significance in multiple comparisons. Symbols represent ns = not significant, * p-value < 0.05, ****p-value < 0.0001.

### CXCR4 and S1P5 are co-expressed in COAD tumors and cooperatively upregulate one another

So far, we have demonstrated that CXCR4 and S1P1/5 trigger converging signaling cascades. Next, we wanted to see whether there is a link between the expression of these receptors. To do this, we analyzed the gene expression of *CXCR4* and *S1PR5* in bulk RNA-seq samples and identified a positive correlation between the two (Fig. 6A). This was confirmed at the protein level by flow cytometry in freshly isolated NK cells (Fig. 6B) and was sustained in cells cultured for 48 h in cytokine regimens (Fig. 6C). Strikingly, incubation of NK cells with CXCL12 lead to an increase in protein levels of S1P5 (Fig. 6D), concomitant with a rise in S1PF binding to NK cells (Fig. 6E). *S1pr5 *expression in mice has been shown to be only partially dependent on the transcription T-bet^43^, suggesting there could be other so far unknown factors related to CXCR4 activation underlying *S1PR5* expression. Although ceralifimod did not influence protein levels of CXCR4 (Fig. 6F), it promoted an increase in CXCR4^+^ cell numbers as measured by flow cytometry (Fig. 6G), suggesting S1P receptor engagement triggers recycling of CXCR4 to the cell surface. We next asked whether simultaneous activation of both receptors resulted in an additive immunosuppressive effect on NK cells. Indeed, the effect observed by ceralifimod and CXCL12 in degranulation in stimulated NK cells was greatly enhanced when both molecules were combined (Fig. 6H). Collectively, our findings suggest that not only CXCR4 and S1P5 exert similar inhibitory effects, but they can amplify desensitization of NK cells by increasing each other’s availability on the cell surface.

CXCL12 and S1P are chemotactic molecules that are upregulated in the tumor microenvironment of multiple malignancies. Colon adenocarcinoma (COAD) stands out as a tumor characterized by particularly high levels of CXCL12 and S1P through SPHK1 activation^44–46^. TCGA data confirmed that *CXCL12* and *SPHK1* expression correlated in COAD tumor samples, but not in normal colon tissue (Fig. 6I), suggesting that ramping up CXCL12 and S1P production by cancer cells in the tumor microenvironment may be one strategy used by cancer cells for tumor progression. In line with our observations, *SPHK1* expression relative to *CXCR4* as well as *CXCL12* relative to *S1PR5* correlated positively in tumor biopsies but not in the respective normal control tissue (Fig. 6J, K). Taken together these data support our hypothesis that engagement of CXCR4 or S1P5 with their cognate ligands creates a positive feedback loop driving one another’s upregulation, in line with our proposed model of S1P1/5 and CXCR4-mediated NK cell desensitization to stimulation by tumor cells (Fig. 6L).

## Discussion

Most of our current knowledge on the role of proteins in NK cell function is based on the clustering of NK cell populations by expression of different markers generally understood to be linked to NK cell characteristics such as maturity, cytotoxicity and exhaustion. Although highly informative, these studies often base their conclusions on NK cell functionality on correlation between genes and reference markers without data supporting functional impact by these subsets. In the present study, we aimed to provide the transcriptional landscape of peripheral NK cells that that are successfully stimulated by tumor cells. We consider this approach has an important added value, as it allows us to avoid any bias stemming from binning NK cells into groups according to pre-established markers. We propose that this degranulating NK cell expression atlas can be used as a powerful resource in combination with existing datasets to predict potential candidates to improve NK cell function and anti-tumor responses or conversely, responses or biomarkers of NK-mediated autoimmune conditions. Additionally, cytokine-based comparisons included in this report may help shed light on the transcriptional state of NK cells in a context-dependent manner; for instance, in NK cells infiltrating highly immunosuppressive environments typically rich in TGF-β, a common occurrence in fibrotic diseases. We also offer an overview of changes in the NK cell transcriptional program upon simultaneous exposure to IL-2 and TGF-β, a slightly more complex system more closely resembling in vivo pathophysiological situations, such as the tumor microenvironment.

Out of all genes revealed to be differentially expressed, we focused our attention on *CXCR4* and *S1PR5*, two of the top three scoring genes in dormant NK cells. CXCR4 is a hepta-helical receptor that plays a critical role during NK cell maturation and acts as an entry receptor for HIV-1^19,47^. During NK cell development, CXCR4 retains immature NK cells in the bone marrow, and is internalized following sustained binding to its ligand, CXCL12. Subsequently, mature NK cells express and present S1P5 at the cell surface, which mediates NK cell egress upon binding to its cognate ligand S1P. *S1PR5 *expression is critical for egress of NK cells from the bone marrow^48^. S1P5 is in fact one of the targets of S1P receptor agonists such as fingolimod (FTY720), a drug approved to reduce aberrant accumulation of lymphocytes in patients suffering from autoimmune diseases such as multiple sclerosis (MS)^49^. Here, we used an S1P1 and S1P5-specific agonist ceralifimod and CXCL12 to study a potential effect by CXCR4 and S1P receptors in NK cell activation complementary to the migratory effect. Treatment of NK cells with CXCL12 or ceralifimod was also linked to poorer outcomes in killing the colon cancer cell line HCT-116. Receptor availability for both molecules in this context was limited: CXCR4 was down-regulated during the 48 h IL-2 pre-stimulation, and S1P receptors, albeit up-regulated upon stimulation with IL-2, were only found in the plasma membrane of approximately 20% of NK cells. The low levels of the receptors in the plasma membrane could underlie the moderate effects observed in the killing assay by CXCL12 and ceralifimod.

CXCR4 and S1P receptors are GPCRs present at the cell surface that once engaged, trigger intracellular signaling cascades downstream of G protein activation. In this study, we have shown that CXCR4 and S1P1/5 receptors can activate Gα_i_-related biological processes including the MAPK pathway and Ca^2+^ influx. Phosphorylation of p42 and store-operated Ca^2+^entry (SOCE) are necessary steps in NK cell degranulation^40,50^. Indeed, they occur together with JNK and STAT5 activation upon activation of the activating receptor Natural Killer Group 2, member D (NKG2D)^51,52^, and downstream of other activating proteins such as NKp30, Nkp44 and NKp46^53^. Small increments in cytosolic Ca^2+^are sufficient to trigger low-grade lytic granule release by NK cells^54^. Indeed, we detected a fraction of NK cells that can undergo degranulation in the absence of stimulation. In this case, only S1PF^+^ NK cells had detectable levels of LAMP1. We speculate that early onset of these events may result in unbalanced vesicle release, leading to changes in polarization and loss of granules ahead of exposure to target tumor cells, resulting in NK cell desensitization.

CXCR4 and S1P5 are sequentially upregulated in the bone marrow during lymphocyte maturation^19^, which may suggest that these proteins are coordinately regulated. Indeed, we uncovered the existence of a crosstalk between these receptors. Activation of S1P1/5 with ceralifimod led to an increase of CXCR4 in the membrane. Additionally, CXCR4 activation with CXCL12 promoted expression of *S1PR5*. This could explain the increase in S1P5 at the cell surface of maturing NK cells after CXCR4 upregulation in the bone marrow^19^. This interplay between the two receptors was also observed in tumor samples. *SPHK1*, and thus S1P production, positively correlated with *CXCR4* expression in COAD tumor samples, but not in normal tissue. *CXCL12* expression correlated with *S1PR5* in the same fashion. On one hand, these data indicate that the presence of S1P and CXCL12 in the tumor can trigger recruitment of NK cells, as only NK cells express *S1PR5 *out of the immune cell repertoire^34^. On the other hand, it raises two non-mutually exclusive possibilities. First, that recruited NK cells co-express *S1PR5* and *CXCR4* and second, that expression of each receptor influences one another. Interestingly, *SPHK1* and *CXCL12 *expression also positively correlated in COAD tumor samples. Tumor cells and other cell types such as cancer-associated fibroblasts or stromal cells in the tumor secrete S1P and CXCL12, which provide them a survival and proliferation advantage^55–59^. These factors can be found in the tumor microenvironment and can be recognized by NK cells to guide them towards the tumor^57,59,60^. Additionally, many tumors are rich in TGF-β, which we show drives the expression of CXCR4, even in the presence of immune stimulatory factors like IL-2. While increased recruitment of NK cells should logically be detrimental for tumor progression, we have shown that activation of CXCR4 and S1P1/5 additively inhibits NK cell activation. Finally, another potential advantage may exist for the tumor in producing CXCL12 and S1P. To reach the solid tumor bed, NK cells must first sense a chemokine gradient, extravasate from the blood, cross the extracellular matrix (ECM) and access the tumor bed. It has been previously reported that blockade of the CXCR4-CXCL12 axis restores migration of immune cells from the fibroblastic towards the juxtatumoral compartment^61^, excluding them from the bulk of the tumor. Interestingly, a recent publication showed that CXCR4 and S1PR1 are among top up-regulated genes in TINKs in colorectal cancer in comparison to circulating cells^62^, and another study showed that *S1pr5* down-regulation is a key marker of tumor-infiltrating NK cells (TINK)^63^. All these findings considered, we speculate that tumor cells benefit from NK cells expressing *CXCR4* and *S1PR5* in two ways: retaining NK cells in the tumor periphery and desensitizing NK cells to stimulation before they reach the tumor stroma.

The role uncovered here for S1PR5 and CXCR4 in NK cell activity validates our RNA-seq data as a valuable resource to uncover new intrinsic modulators of NK cell activity. Due to their nature as druggable membrane receptors, CXCR4, S1P1 and S1P5, they might be exploited in potential cancer therapies, as illustrated by the current availability of drugs in the clinic. Future studies will however be required to explore the role of these receptors in vivo to understand the intricacies of each function orchestrated by *CXCR4* and *S1PR5* in tumor-infiltrating NK cells.

## Methods

### Cell lines

Colon carcinoma HCT-116 (CCL-247) cells were purchased from the American Type Culture Collection (ATCC) and gliosarcoma SF-539 (CVCL_1691) cells were purchased from the National Cancer Institute (NCI). HCT-116 cells cultured in McCoy 5 A Medium (gibco, 16600082) supplemented with 10% Heat-inactivated (HI) Sera (gibco, A5256801). SF-539 cells were cultured in RPMI 1640 GlutaMAX medium (Gibco, 61870036) supplemented with 10% HI Sera. Cells were incubated at 37 °C in a 5% CO_2_ environment. Standardized qPCR tests confirmed absence of mycoplasma in cell lines. Cell lines were authenticated by STR profiling.

### Isolation and in vitro stimulation of primary human immune cells

Primary human immune cells were obtained from residuals of leukoreduction from blood donors. All human samples used for the conduction of this study were obtained from DRK (German Red Cross)-Blutspendedienst Baden-Württemberg–Hessen gemeinnützige GmbH, who has collected all human samples under a valid informed consent obtained from all donors as well as according to all relevant guidelines and regulations. All in vitro experimental procedures were approved by the University of Ulm ethical committee. Peripheral blood mononuclear cells (PBMCs) were isolated using Ficoll-Plaque (Cytiva, 17144003) in SepMate Tubes (STEMCELL, 85450). Red blood cells were lysed using RBC lysis buffer (eBioscience, 00-4333-57). For NK cell isolation the EasySep Human NK Cell Isolation Kit (STEMCELL, 17955RF) was used. NK cells were cultured immediately after isolation in IMDM media (gibco, 12440053) supplemented with 10% HI Sera. In all instances, NK cells were kept in culture for 48 h before being used for experiments. To modulate NK cell activity, NK cells were either stimulated with recombinant Human IL-2 10 ng/mL (BioLegend, 589104), or inhibited with TGF-β (Bio-Techne, 7754-BH-005/CF) at the same concentration, and cells were cultured at 37 °C and 5% CO_2_ for 2 days. If ceralifimod (Merk, SML3668) or S1P5 inhibitor (S1P5i) were used, they were added to the media throughout the culture at a concentration of 1 µM. For T cell culture, CD8^+^ T cells were freshly isolated from PBMCs using the CD8 + T Cell Isolation Kit (Miltenyi, 130-096-495). Isolated CD8^+^ T cells were seeded in RPMI 1640 GlutaMAX medium supplemented with 10% HI Sera and 50 µM 2-Mercaptoethanol (Gibco, 31350010) in the presence of 10 ng/mL IL-2 in plates pre-coated with Anti-Human CD28 (Thermo Fisher, 16-0037-85) and Anti-Human CD3 (Thermo Fisher, 16-0289-85) antibodies at 1 µg/mL concentrations for pre-stimulation. The day after cells were collected and transferred to non-coated plates. NK and CD8^+^ T cells were treated overnight with CXCL12 (BioLegend, 581202) and plerixafor (Selleckchem, S8030) at 100 ng/mL and 10 µM concentration respectively.

### Assessment of degranulation and CTL activation by flow cytometry

NK cells were seeded in fresh media in the presence of 1 µL Anti-Human CD107a antibody (BD, 555801) per 100,000 cells with HCT-116 or SF-539 cells at a 1:1 target to effector (T:E) ratio. Cells were co-cultured for 2 h, and then stained with antibodies. CD107a antibody was used to monitor degranulation, Anti-Human CD56 (BD Biosciences, 562751), CD3e (BD Biosciences, 558257) and CD45 (Miltenyi, 130-113-123) were used to distinguish NK cells. Cells were additionally stained with Anti-Human CXCR4 (BD Biosciences, 555976) and Anti- Human S1PR1 (eBioscience, 50-3639-42) for NK cell phenotyping. S1P-fluorescein (S1PF) (Echelon Biosciences, S-200 F) was used as a pan-marker of S1PR proteins. CD8^+^ T cells pre-stimulated with Anti-Human CD3e and CD28 antibodies were re-stimulated with in Anti-Human CD3e antibody pre-coated plates for 2 h in the presence of CD107a antibody at the same ratio as NK cells. T cells were then collected and further stained with Anti-CD107a, CXCR4, S1PR1 and Anti-Human CD8 (BD Biosciences, 562428) antibodies and S1PF to identify degranulating CD8^+^ T cells. Fixable Viability Dye eFluor 780 (eBioscience, 65-0865-14) was used in all instances to distinguish viable cells. Cells were analyzed using the LSR Fortessa Analyzer (BD Biosciences). Analysis of flow cytometry data was performed using FlowJo v10.

### Sample sort and RNA preparation

Stained NK and HCT-116 pools were sorted using the BD FACSAria III Sorter. 40,000 cells per condition were sorted into DNA LoBind Tubes (eppendorf, 0030108501) containing a small volume of PBS. Cells were centrifuged at 500 g for 10 min, resuspended, mixed thoroughly in QIAzol Lysis Reagent (Qiagen, 79306) and frozen at −80 °C. For RNA extraction, samples were transferred to PRIME PhaseLock tubes (QuantaBio, 2302820) and chloroform (Honeywell, C2432-25mL) was added at a 5:1 ratio and vigorously mixed. Samples were centrifuged. The resulting top aqueous layer was mixed with 1.5 volumes of Ethanol 100% (Roth, 9065.3). Samples were then pipetted onto miRNeasy spin columns included in the miRNease Micro Kit (Qiagen, 217084). Next steps were performed as suggested in the protocol provided by the manufacturer. DNase I (Agilent, 600031) was used to digest DNA present in the RNA samples. RNA was eluted with 14 µL UltraPure RNAse-free water in Lo-Bind tubes and stored at − 80 °C.

### Low input RNA-seq

For bulk transcriptomic profiling of FACS isolated cells we conducted low input RNA-seq with the SMART-seq v4 and Illumina DNA prep kits. Briefly, total RNA samples were quantitatively and qualitatively assessed using the DNF-472 HS RNA Kit on a 96-channel Fragment Analyzer (Agilent). All Total RNA samples had a RIN > 7.5. A total RNA input of 4ng was employed for cDNA synthesis with the SMART-seq^®^ v4 Ultra Low Input RNA kit with 13 amplification cycles as per manufacturer’s instructions (Takara Bio, #634891). Following cDNA clean-up, 10 ng of cDNA was used to generate final sequencing libraries with the tagmentation-based Illumina DNA prep kit (Illumina, #20018705) and the IDT^®^DNA/RNA Indexes set A (Illumina, #20027213). Indexing PCR was performed with 8 cycles and final low-input mRNA-seq libraries eluted in 22 µL of EB Buffer (Qiagen). Final sequencing libraries were quantified by the High Sensitivity dsDNA Quanti-iT Assay Kit (Thermo Fisher) on a Synergy HTX (BioTek). Libraries were assessed for size distribution and adapter dimer presence by the High Sensitivity NGS DNF-474 Kit on a 96-channel Fragment Analyzer (Agilent). Sequencing libraries were normalized on a MicroLab STAR (Hamilton), pooled, and subsequently sequenced on an Illumina Novaseq 6000 (Read Parameter: Rd1: 101 bp, Rd2: 10 bp, Rd3: 10 bp, Rd4:101 bp) with an average depth of ~ 44 million Pass-Filter reads per sample.

### RNA-seq analysis

The bulk RNA-Seq analysis pipeline was executed as described before^64^. In brief, reads passing quality control filter were mapped against GRCh38 using STAR (v2.5.2b) aligner. Gene expression levels were quantified using RSEM (v1.3.0) and featureCounts (v1.5.1). For quality control FastQC (v0.11.5), picardmetrics (v0.2.4) and dupRadar (v1.2.2) were employed.

Differential expression testing was performed using the topTags() function with *n*= 20,000 from the edgeR R Bioconductor package^65^, after the expression matrix was filtered for protein-coding genes. The following contrasts were used:

- *LAMP_high_vs_low_in_PBS*: control_LAMP1^hi^ - control_LAMP1.
- *Hostile_degranulation*: (TGF-treated_LAMP^hi^) - (TGF-treated_LAMP^−^ + control_LAMP^hi^ + control_LAMP^−^)/3.
- *IL2_vs_PBS_in_LAMP1_high*: IL2-treated_LAMP1^hi^ - control_LAMP1^+^.

The results were visualized in volcano plots, labeling the top 50 genes based on the lowest mean of the ranks of -log_10_(p-adjusted) and fold change.

### Signaling pathway impact analysis

To identify enriched pathways, differentially expressed genes were used as input to the SPIA() function from the CBDD package (Clarivate, London, UK)^66^ using the following parameters: lfc_cutoff = 1, fdr_cutoff = 0.01, and nb = 2000. Metabase (Clarivate, London, UK) was used as the reference pathway database and the top 100 pathways were selected based on the lowest mean of the ranks of − log_10_(p-adjusted) and absolute perturbation values. These pathways were subsequently plotted in a dot plot, showing the different contrasts from which the differentially expressed genes derived as indicated side by side.

### Tumor cell killing assays

HCT-116 and SF-539 were used to assess tumor cell killing. For NK cell-dependent killing, tumor cells were seeded in 96-well plates in triplicates, followed by addition of pre-stimulated or non-pre-stimulated NK cells at increasing T:E ratios. For the EpCAM-based CD8^+^ T cell killing, CD8^+^T cells were freshly isolated from PBMCs and seeded in T cell media in the presence or absence of treatment. After 48 h they were added to tumor cells seeded in 96 well plates in concentrations of EpCAM T cell engager (TcE) ranging from 0 to 100 fM. EpCAM TcE was expressed in-house following a previously published sequence^67^. At the end of the culture half of the supernatant volume was collected for further assays. The remaining material was used to assess cell viability using CellTiter-Glo reagents (Promega, G7571) following the manufacturer’s suggested protocol. Extent of NK cell and T cell killing was defined.

### Synthesis of S1P5 inhibitor

Synthesis of the S1P5 inhibitor “compound 15” was performed as previously reported^36^.

### Detection of S1P, CXCL12 and IFNγ by ELISA

Media was collected from NK cell cultures. Samples were centrifuged at 6000 g for 10 min, and supernatants were frozen at −80 °C until further use. For S1P ELISA, the Human Sphingosine 1 Phosphate ELISA Kit (Biorbyt, orb563906) was used. For IFN-γ detection, the IFN gamma Human ELISA Kit (Thermo Fisher, EHIFNG) was utilized. For CXCL12 detection, the Human SDF-1 alpha/CXCL12A ELISA Kit was used (Thermo Fisher Scientific, EHCXCL12A). ELISAs were conducted following the manufacturer recommendations. Supernatants were diluted 1:4 in assay buffer before use.

### Detection of protein in cell lysates by Peggysue

Cells were centrifuged at 500 g for 5 min, washed once with PBS, and then resuspended in RIPA buffer (Thermo Scientific, 89900) supplemented with Protease and Phosphatase inhibitor cocktail (Thermo Scientific, 1861281). Samples were mixed thoroughly and incubated on ice for 30 min, then centrifuged at 16,000 g for 10 min to separate protein solution from cell debris. Protein levels were measured using the BCA system (Thermo Fisher, 23225). Protein immunoblot was run in the PeggySue (BioTechne, 004-800), an automated immunoblot system in which protein separation is performed in a capillary instead of a SDS polyacrylamide gel. 3 µg of each sample were pipetted into the PeggySue plate, together with blocking antibody diluent, primary and secondary antibodies, and peroxidase mixed with luminol. The run was performed following the manufacturer instructions. Anti-Human S1PR5 (Proteintech, 13874-1-AP), CXCR4 (Thermo Fisher Scientific, 35–800), SPHK1 (NeoBiotech, NB-22-20477), SPHK2 (GeneTex, GTX105152), P-SPHK1 (Proteintech, 19561-1-AP), P-SPHK2 (NeoBiotech, NB-22-1268), GNAI2 (abcam, ab157204), p44/42 (Cell Signaling, 9102 S), P-p44/p42 (Cell Signaling, 4370 S), cofilin (Cell signaling, 5175 S) and Anti-FLAG Tag (Thermo Fisher Scientific, MA1-91878) antibodies were used. Quantification of samples was performed using the Compass for SW software.

### Transfection of HCT-116

Sequences used for S1P1 and S1P5 plasmid preparation were corresponding to canonical variants of each protein, P21453 and Q9H228-1, respectively. Both S1P1 and S1P5 plasmids were designed in the GeneArt tool (Thermo Fisher Scientific) containing an N-terminal Flag tag (DYKDDDDK) to facilitate detection. HCT-116 cells were seeded one day before transfection in full HCT-116 medium at a 30% confluence. Cells were transfected with empty vector (mock), S1P1 or S1P5 plasmids using Lipofectamine 3000 (Thermo Fisher, L3000008), following the manufacturer protocol. After 24 h, transfection levels were assessed by flow cytometry using anti-FLAG antibody (BioLegend, 637322). Cells were also lysed and used for immunoblotting experiments as indicated in the previous section.

### Migration assay

In vitro assessment of NK cell migration was performed using polycarbonate membrane Transwell plates with a 6.5 mm diameter and 5 μm pore size (Corning Costar, #3421). Briefly, 1.25 × 105 NK cells were treated for 2 h with 10 µM plerixafor. Then, untreated or treated NK cells were seeded in the upper chamber of the well in 200 µL of fresh NK cell medium. In the lower chamber, HCT-116 cells were seeded in NK cell medium in the presence or absence of CXCL12 at a 10 ng/mL concentration. E: T ratio was kept at 1.25:1. After 2 h of culture at 37 °C and 5% CO_2_, media in the lower chamber was harvested and migrated cells were quantified using flow cytometry analysis.

### Ca^2+^ analysis with Fluo-3

5 × 10^5^ NK or CD8 + T cells were seeded the day before the assay with IL-2 at a concentration of 10 ng/mL in the presence of drugs or DMSO. The day of the experiment cells were cultured at 37 °C with pre-warmed serum-free RPMI 1640 containing 2 µM of Fluo-3 AM (Thermo Fisher, F1242) staining solution. At the end of incubation cells were washed twice with serum-free RPMI 1640. Finally, cells were resuspended in PBS buffer supplemented with 2% FBS for analysis. Fluo-3 signal was detected using the LSR Fortessa Analyzer in the medium speed setting for 30 s.

### Statistical analysis

Two-tailed paired t-student tests were performed to compare groups in simple tests. Paired-sample One-way ANOVA or Two-way ANOVA were used to assess statistically significant differences between groups when one variable or two variables were assessed respectively. Statistical significance was defined as following: ns = not significant, *p-value < 0.05, **p-value < 0.01, ***p-value < 0.001, ****p-value < 0.0001. Error bars were plotted according to N and SD values. For statistical analysis of RNA-seq results refer to RNA-seq analysis method description.

## Electronic supplementary material

Below is the link to the electronic supplementary material.

Supplementary Material 1.

Supplementary Material 2.

Supplementary Material 3.

## Data Availability

The dataset has been reported to GEO (GSE284000) and are are available upon request from the corresponding author.
