# Supplementary material for: Transcriptional signature of rapidly responding NK cells reveals S1P5 and CXCR4 as anti-tumor response disruptors
Source: Sci Rep. 2025 Mar 28;15:10769. doi: 10.1038/s41598-025-95211-7 (PMC11953373; doi:10.1038/s41598-025-95211-7)
Supplement: Supplementary file 3 — Supplementary Material 3. [file 41598_2025_95211_MOESM3_ESM.docx]

# **Supplementary information**


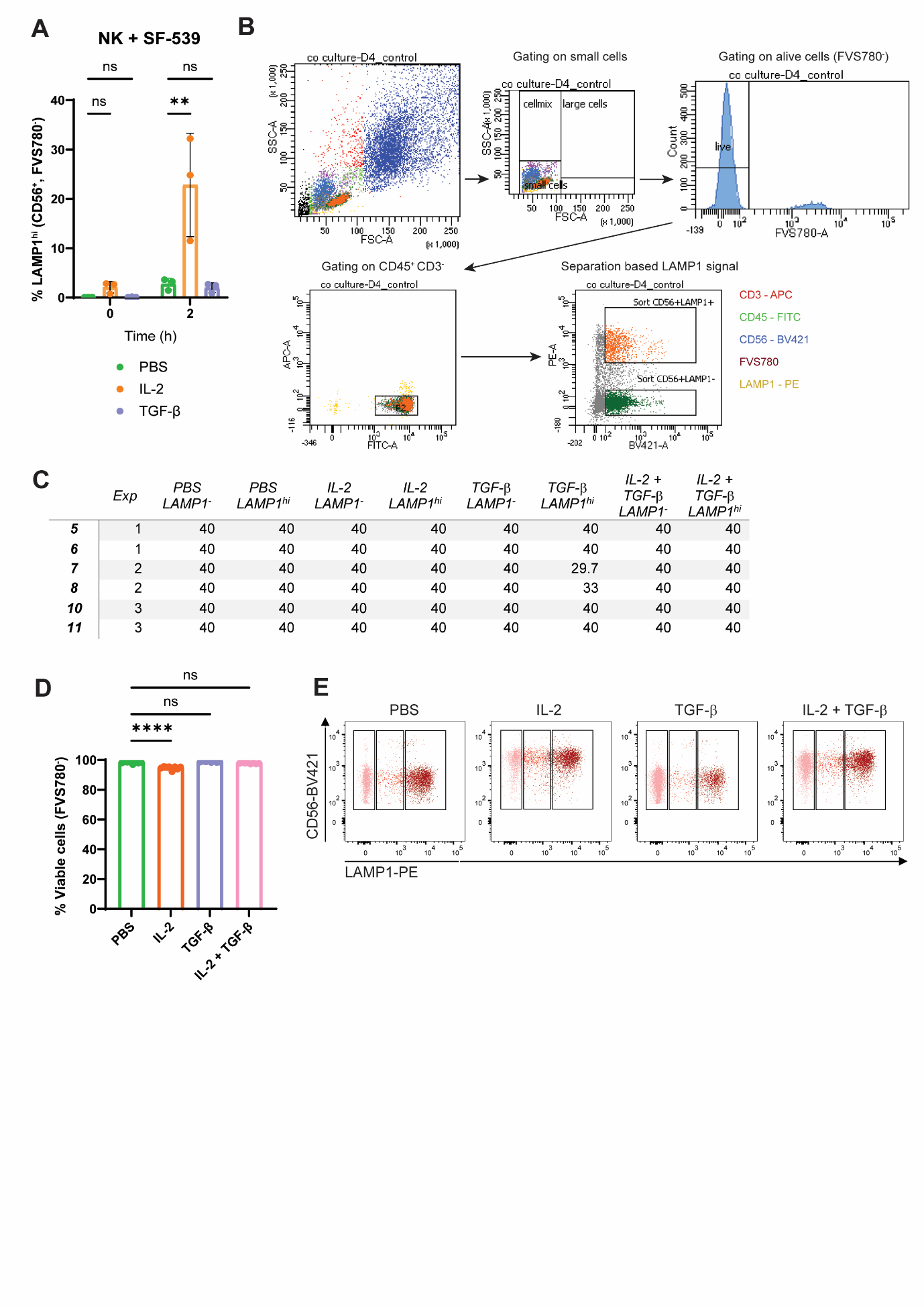


**Supplementary Figure S1. Identification of LAMP1^hi^ cells upon treatment with cytokines.** In all instances NK cells were treated with the following cytokine concentrations: 20 ng/mL IL-2, 10 ng/mL TGF-β, a combination of IL-2 and TGF-β or vehicle (PBS). **A**) Percentage of LAMP1^+^ in NK cells co-cultured for 2 hours with SF-539 or left unstimulated. **B**) Gating strategy used for the selection of LAMP1^hi^ and LAMP1^-^ NK cells for RNA-seq sample preparation. Briefly, small cells were gated to discard most of HCT-116 cells (large cells). Then, a gate was made on living cells (FVS780^-^). Next, CD45^+^CD3^-^ were gated to ensure selection of immune cells and rule out NKT cells. Finally, gates were made to sort CD56^+^LAMP1^-^ cells and highly positive CD56^+^LAMP1^+^ cells (LAMP1^hi^). **C**) Numbers of isolated NK cells for each donor used in the RNA-seq. Values represent number of thousands of cells. **D**) Percentage of viability of NK cells after 48 hours of treatment with cytokines measured using FVS780 stain. **E**) Flow cytometry plots of LAMP1 signal in cytokine-treated NK cells after stimulation with HCT-116 for 2 hours (RNA-seq samples). For all charts, every data point corresponds to the average value in each independent donor and bars the mean ± SD. One-way ANOVA statistical tests were used in systems with several conditions for a single variable (**A**) and Dunnett’s test was used to perform multiple comparisons. Symbols represent ns = not significant, ** p-value < 0.01.


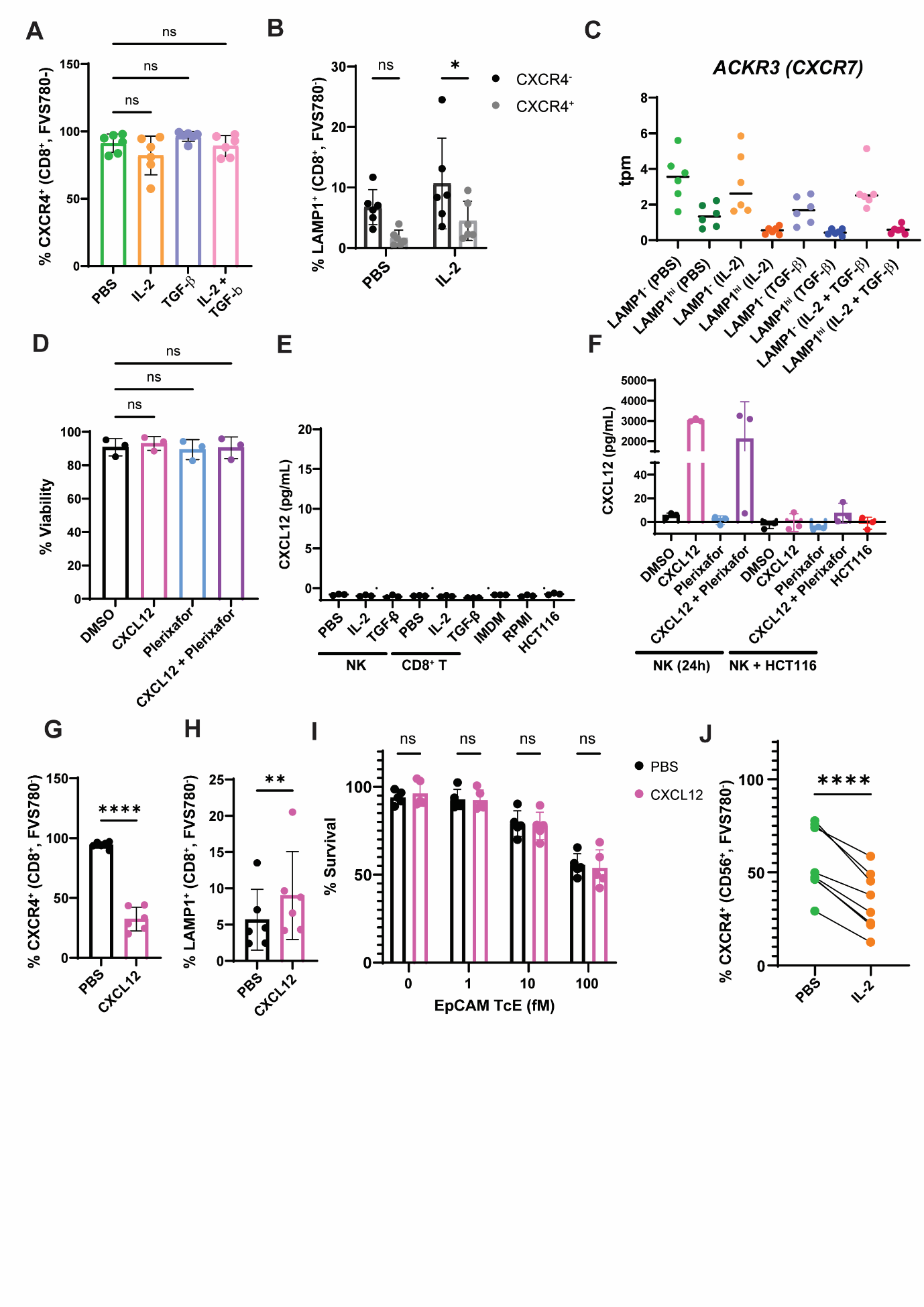


**Supplementary Figure S2. CXCR4 activation does not hinder peripheral CD8^+^ T cell activity**. **A** Percentage of CXCR4^+^ cells measured by flow cytometry upon treatment with cytokines: 10 ng/mL IL-2, 10 ng/mL TGF-β, combination or vehicle (PBS). **B**) Percentage of LAMP1^+^ cells in the CXCR4^-^ and CXCR4^+^ subsets. **C**) Expression levels of *ACKR3* in the samples included in the RNA-seq quantified in transcripts per million (tpm). **D**) Viability of NK cells upon treatment with 100 ng/mL CXCL12, 10 µM plerixafor or CXCL12 + plerixafor overnight. Viability was measured as % of FVS780^-^ cells. **E**) CXCL12 ELISA of supernatants of NK, CD8^+^ T and HCT-116 mono-cultures. Supernatants were collected after 48 hours. **F**) CXCL12 ELISA of supernatants from NK cells monocultures and 24 hour co-cultures with HCT-116 cells after exposure to CXCL12, plerixafor or CXCL12 + plerixafor. **G**) Quantification of CXCR4^+^ CD8^+^ T cells following overnight incubation with 100 ng/mL CXCL12 or PBS. **H**) Quantification of LAMP1^+^ CD8^+^ T cells. Cells were pre-stimulated with 1 µg/mL Anti-Human CD3ε + Anti-Human CD28 antibodies overnight, then cultured in uncoated plates in the presence of 100 ng/mL CXCL12 or PBS. Degranulation was induced by re-plating in Anti-Human CD3ε-coated plates, full staining was performed after 2 hours. Changes in HCT-116 survival in co-cultures with CD8^+^T cells (**I**) or NK cells (**J**) pre-incubated with CXCL12 or PBS, supplemented with increasing levels of EpCAM TcE. For all charts, every data point corresponds to the average value in each independent donor and bars the mean ± SD. T student tests were used for comparisons between two conditions (**G**, **H**, **J**). One-way ANOVA statistical tests were used in systems with several conditions for a single variable (**A**, **D**). For these, Dunnett’s test (**A**) or Šídák’s test (**D**) were used to perform multiple comparisons. The Two-way ANOVA statistical test was performed for analysis containing two variables (**A**, **H**), for which Šídák’s test for multiple comparisons was used. Symbols represent ns = not significant, * p-value < 0.05, ** p-value < 0.01, **** p-value < 0.0001.


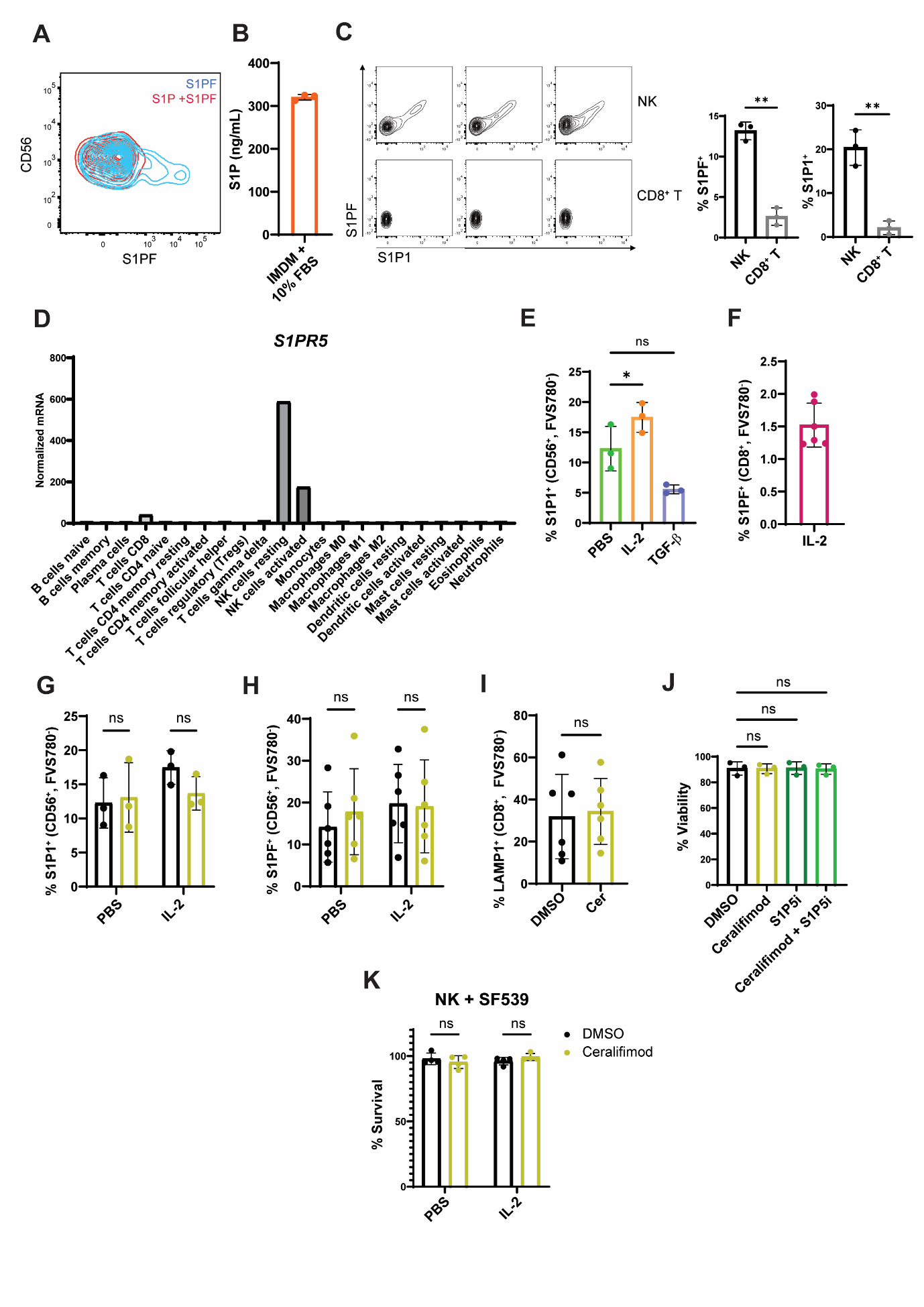


**Supplementary Figure S3. Peripheral CD8^+^ T cells lack S1P receptors in the plasma membrane.** **A**) Flow cytometry chart of 10 µM S1PF binding to NK cells in resting and cells blocked with 3.25 µM S1P for 30 minutes at 4°C. Representative image of one out of three independent donors. **B**) S1P ELISA of media used for NK cell culture. **C**) Flow cytometry charts and quantification of Anti-S1P1 antibody and S1PF binding to freshly isolated NK and CD8+ T from three independent donors. **D**) Normalized expression levels (Affymetrix intensity) of *S1PR5* in immune cell populations. Data was extracted from Newman. AM, *et al.*, 2015^21^. **E**) Percentage of S1P1^+^ in NK cells treated for 48 hours with 10 ng/mL IL-2, 10 ng/mL TGF-β or vehicle. **F**) Quantification of CD8^+^ T cells treated with IL-2 that bind S1PF. Percentage of S1P1^+^ (**G**) and S1PF^+^ (**H**) NK cells upon treatment with vehicle, 10 ng/mL IL-2 and 1 µM ceralifimod for 48 hours. **I**) Quantification of LAMP1^+^CD8^+^ T cells upon incubation with vehicle or 1 µM ceralifimod. Cells were pre-stimulated with 1 µg/mL Anti-Human CD3ε + Anti-Human CD28 antibodies overnight, left to rest for 1 day and further cultured for 2 hours in plates pre-coated with Anti-CD3ε antibody. **J**) Percentage of FVS780^-^ NK cells upon treatment with 1 µM ceralifimod, 1 µM S1P5i or ceralifimod + S1P5i overnight. **K**) Survival of SF-539 cells after 24 hours of co-culturing with NK cells previously treated with indicated regimens at different E:T ratios. For all charts, every data point corresponds to the average value in each independent donor and bars the mean ± SD. T student tests were used for comparisons between two conditions (**C**, **I**). One-way ANOVA statistical tests were used in systems with several conditions for a single variable (**E**, **J**). For these, Dunnett’s test (**E**) or Tukey’s test (**J**) were used to perform multiple comparisons. The Two-way ANOVA statistical test was performed for analysis containing two variables (**G**, **H**, **K**), for which Šídák’s test for multiple comparisons was used. Symbols represent ns = not significant, * p-value < 0.05, ** p-value < 0.01.

| Gene ID | Symbol | Biotype | log2FoldChange  CTRL_LAMP1^hi^ vs CTRL_LAMP1^-^ | adj.p-value  CTRL_LAMP1^hi^ vs CTRL_LAMP1^-^ |
| --- | --- | --- | --- | --- |
| ENSG00000265972 | *TXNIP* | protein_coding | -2.51 | 1.27E-13 |
| ENSG00000163462 | *TRIM46* | protein_coding | -2.31 | 0.00283 |
| ENSG00000121966 | *CXCR4* | protein_coding | -2.22 | 3.67E-05 |
| ENSG00000164106 | *SCRG1* | protein_coding | -2.17 | 0.00223 |
| ENSG00000104081 | *BMF* | protein_coding | -2.15 | 0.000178 |
| ENSG00000180739 | *S1PR5* | protein_coding | -2.09 | 4.29E-18 |
| ENSG00000170289 | *CNGB3* | protein_coding | -2.08 | 0.0293 |
| ENSG00000165309 | *ARMC3* | protein_coding | -2.04 | 0.0367 |
| ENSG00000166289 | *PLEKHF1* | protein_coding | -1.97 | 9.69E-13 |
| ENSG00000132010 | *ZNF20* | protein_coding | -1.94 | 0.0357 |
| ENSG00000119922 | *IFIT2* | protein_coding | -1.93 | 1.05E-08 |
| ENSG00000177875 | *CCDC184* | protein_coding | -1.92 | 0.0443 |
| ENSG00000136630 | *HLX* | protein_coding | -1.92 | 0.048 |
| ENSG00000205209 | *SCGB2B2* | protein_coding | -1.91 | 0.011 |
| ENSG00000197587 | *DMBX1* | protein_coding | -1.87 | 0.0241 |
| ENSG00000166707 | *ZCCHC18* | protein_coding | -1.86 | 0.0315 |
| ENSG00000109163 | *GNRHR* | protein_coding | -1.86 | 0.0379 |
| ENSG00000133742 | *CA1* | protein_coding | -1.84 | 0.035 |
| ENSG00000183662 | *FAM19A1* | protein_coding | -1.82 | 0.0143 |
| ENSG00000075886 | *TUBA3D* | protein_coding | -1.82 | 0.0177 |
| ENSG00000108950 | *FAM20A* | protein_coding | -1.81 | 0.00278 |
| ENSG00000157514 | *TSC22D3* | protein_coding | -1.8 | 1.19E-05 |
| ENSG00000176749 | *CDK5R1* | protein_coding | -1.77 | 7.12E-09 |
| ENSG00000166793 | *YPEL4* | protein_coding | -1.77 | 0.0283 |
| ENSG00000243284 | *VSIG8* | protein_coding | -1.76 | 0.0176 |
| ENSG00000125910 | *S1PR4* | protein_coding | -1.75 | 3.08E-10 |
| ENSG00000108576 | *SLC6A4* | protein_coding | -1.71 | 0.00113 |
| ENSG00000078053 | *AMPH* | protein_coding | -1.7 | 3.17E-05 |
| ENSG00000267508 | *ZNF285* | protein_coding | -1.67 | 0.00307 |
| ENSG00000275793 | *RIMBP3* | protein_coding | -1.67 | 0.0395 |
| ENSG00000103942 | *HOMER2* | protein_coding | -1.66 | 4.35E-08 |
| ENSG00000172602 | *RND1* | protein_coding | -1.66 | 0.0397 |
| ENSG00000006555 | *TTC22* | protein_coding | -1.62 | 1.61E-06 |
| ENSG00000196659 | *TTC30B* | protein_coding | -1.62 | 0.0177 |
| ENSG00000172345 | *STARD5* | protein_coding | -1.56 | 6.75E-10 |
| ENSG00000213658 | *LAT* | protein_coding | -1.54 | 1.69E-08 |
| ENSG00000131634 | *TMEM204* | protein_coding | -1.54 | 0.00215 |
| ENSG00000185669 | *SNAI3* | protein_coding | -1.53 | 1.25E-07 |
| ENSG00000213085 | *CFAP45* | protein_coding | -1.53 | 0.0465 |
| ENSG00000163545 | *NUAK2* | protein_coding | -1.51 | 0.000394 |
| ENSG00000102032 | *RENBP* | protein_coding | -1.5 | 0.000469 |
| ENSG00000214872 | *SMTNL1* | protein_coding | -1.47 | 0.00105 |
| ENSG00000084110 | *HAL* | protein_coding | -1.46 | 0.00891 |
| ENSG00000197933 | *ZNF823* | protein_coding | -1.46 | 0.0373 |
| ENSG00000171094 | *ALK* | protein_coding | -1.45 | 0.0341 |
| ENSG00000273045 | *C2orf15* | protein_coding | -1.43 | 0.00929 |
| ENSG00000170379 | *TCAF2* | protein_coding | -1.42 | 4.33E-07 |
| ENSG00000089163 | *SIRT4* | protein_coding | -1.42 | 0.0146 |
| ENSG00000103343 | *ZNF174* | protein_coding | -1.41 | 3.23E-05 |
| ENSG00000165879 | *FRAT1* | protein_coding | -1.41 | 4.72E-05 |

**Supplementary Table 1.** Top 50 down-regulated genes in LAMP1^hi^ cells in comparison to LAMP1^-^ cells in the control condition (PBS) defined by log2Fold change and p-value.
